# Synergistic Reaction of SO_2_ with NO_2_ in Presence of H_2_O and NH_3_: A Potential Source of Sulfate Aerosol

**DOI:** 10.3390/ijms20153746

**Published:** 2019-07-31

**Authors:** Zehua Wang, Chenxi Zhang, Guochun Lv, Xiaomin Sun, Ning Wang, Zhiqiang Li

**Affiliations:** 1 Environment Research Institute, Shandong University, Jinan 250100, China; 2 College of Biological and Environmental Engineering, Binzhou University, Binzhou 256600, China; 3 Center for Optics Research and Engineering (CORE), Shandong University, Qingdao 266237, China

**Keywords:** sulfuric acid, sulfur dioxide, nitrogen dioxide, synergistic oxidation, hydrolysis reaction

## Abstract

Effect of H_2_O and NH_3_ on the synergistic oxidation reaction of SO_2_ and NO_2_ is investigated by theoretical calculation using the molecule system SO_2_-2NO_2_-nH_2_O (*n* = 0, 1, 2, 3) and SO_2_-2NO_2_-nH_2_O-mNH_3_ (*n* = 0, 1, 2; *m* = 1, 2). Calculated results show that SO_2_ is oxidized to SO_3_ by N_2_O_4_ intermediate. The additional H_2_O in the systems can reduce the energy barrier of oxidation step. The increasing number of H_2_O molecules in the systems enhances the effect and promotes the production of HONO. When the proportion of H_2_O to NH_3_ is 1:1, with NH_3_ included in the system, the energy barrier is lower than two pure H_2_O molecules in the oxidation step. The present study indicates that the H_2_O and NH_3_ have thermodynamic effects on promoting the oxidation reaction of SO_2_ and NO_2_, and NH_3_ has a more significant role in stabilizing product complexes. In these hydrolysis reactions, nethermost barrier energy (0.29 kcal/mol) can be found in the system SO_2_-2NO_2_-H_2_O. It is obvious that the production of HONO is energetically favorable. A new reaction mechanism about SO_2_ oxidation in the atmosphere is proposed, which can provide guidance for the further study of aerosol surface reactions.

## 1. Introduction

Sulfur dioxide (SO_2_), a major air pollutant, is released into the atmosphere via the combustion of sulfur containing fuels and industrial production [1,2,3], volcanic eruptions, and decomposition of sulfides in nature. SO_2_ can be oxidized to form sulfuric acid (H_2_SO_4_) through the homogeneous or heterogeneous processes [4,5,6], which include gas-phase oxidation by hydroxyl radical (OH•) and in-cloud oxidation by dissolved ozone (O_3_) and hydrogen peroxide (H_2_O_2_) [7,8,9]. It has been proved that sulfuric acid is the main contributor to the new particle formation, resulting in serious environmental issues such as haze and acid rain [10,11,12]. Thus, it is significant to investigate the SO_2_ oxidation mechanism in the atmosphere.

In hazy weather, researchers have found that a considerable amount of PM_2.5_ can be formed in the atmosphere, and sulfate is the significant component of the fine particulate matter [13,14,15,16,17,18]. Previous studies have indicated that, as the haze weather gets worse, the concentration of O_3_ decreases, while the concentrations of NO_2_ and sulfate increase [19]. However, widespread formation pathways (gas-phase oxidation and in-cloud oxidation) of sulfate cannot account for the phenomena of high content of sulfate. Some researchers have investigated that the oxidation reaction of SO_2_ by NO_2_ is proposed as a missing key pathway to form sulfate in a special condition [19,20,21,22,23,24]. Wang et al. have considered that NO_2_ could effectively react with SO_2_ in the presence of H_2_O and NH_3_ during the severe haze period [25]:

(1) $${\mathrm{SO}}_{2}\left(g\right)+2{\mathrm{NO}}_{2}\left(g\right)+2H_{2}O\left(\mathrm{aq}\right)\to 2H^{+}\left(\mathrm{aq}\right)+{\mathrm{SO}}_{4}{}^{2-}\left(\mathrm{aq}\right)+2\mathrm{HONO}\left(g\right)$$

(2) $$2{\mathrm{NH}}_{3}\left(g\right)+{\mathrm{SO}}_{2}\left(g\right)+2{\mathrm{NO}}_{2}\left(g\right)+2H_{2}O\left(\mathrm{aq}\right)\to 2{\mathrm{NH}}_{4}{}^{+}\left(\mathrm{aq}\right)+{\mathrm{SO}}_{4}{}^{2-}\left(\mathrm{aq}\right)+2\mathrm{HONO}\left(g\right)$$

From Reaction (1) and (2), it can be seen that the final products primarily include SO_4_^2−^ and HONO.

In the presence of water, the process of NO_2_ dimerization is likely to occur in polluted atmospheric conditions [26,27]. Some studies have demonstrated that N_2_O_4_, the dimer of NO_2_, serves as the oxidant to oxidize SO_2_ on the aerosol surface [28,29,30]. For NO_2_ dimer, three isomers, symmetric sys-O_2_N-NO_2_, and asymmetric t-ONONO_2_, c-ONONO_2_, can be found. The sys-O_2_N-NO_2_ is very stable due to its symmetry, while asymmetric ONO-NO_2_ can be rapidly converted to NO^+^ and NO_3_^−^ in the presence of water [31,32,33,34]. The ion pairs (NO^+^NO_3_^−^) can also oxidize many organic and inorganic compounds [35]. Although these studies have shown that SO_2_ can react with NO_2_ to produce H_2_SO_4_, the reaction mechanism is not completely understood.

In this paper, we researched the gas-phase reaction of SO_2_ with NO_2_ using the density functional theory (DFT) method. The different number of water molecules is added to the reaction so as to investigate the role of water molecules in the oxidation reaction. In addition, the effect of NH_3_ in the oxidation reaction also is considered.

## 2. Results and Discussion

### 2.1. The Reaction of SO_2_-2NO_2_-nH_2_O (n = 0, 1, 2, 3)

#### 2.1.1. The Reaction of SO_2_-2NO_2_

**Figure 1:**
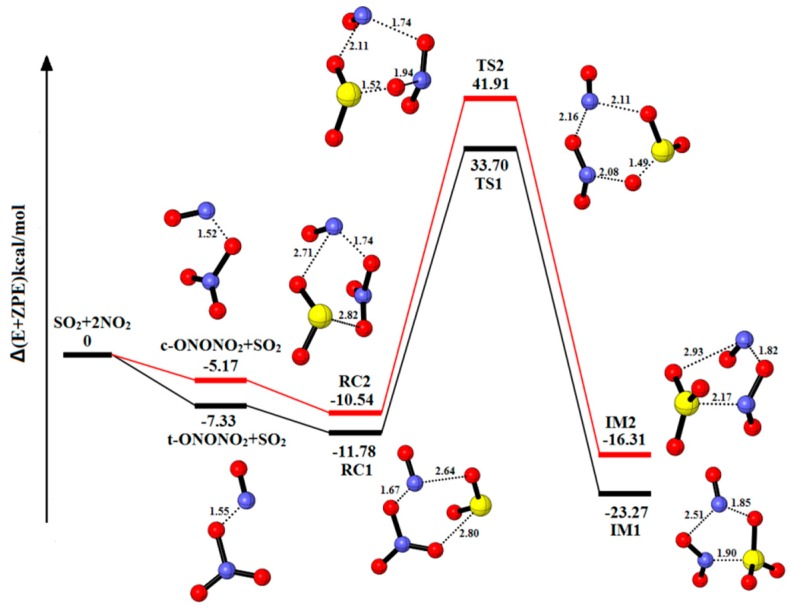
Potential energy profile for the reaction of SO_2_-2NO_2_. The yellow spheres are S atoms, the red spheres are O atom, and the blue spheres are N atom, respectively. (Unit: Energies in kcal/mol; bond lengths in angstroms). The black and red lines are the different pathways. The black line represents the reaction of t-ONONO_2_-SO_2_, and the red line is the reaction of c-ONONO_2_-SO_2_.

In the absence of H_2_O, the equilibrium structures and potential energy are shown in Figure 1, Table 1 and Table 2 present the corresponding energy data and the numerical value of charge distribution of NO^+^NO_3_^−^ and NO^+^NO_2_^−^. The relative energies, enthalpies and Gibbs free energies in all relevant species for the system SO_2_-2NO_2_ are summarized in Table S1. Two main pathways are found due to the formation of two NO_2_ dimers, t-ONONO_2_ and c-ONONO_2_. The pre-reactive complexes (RC1 and RC2) are produced by a collision of SO_2_. The IM1 (a binding of −23.27 kcal/mol) is formed via transient state (TS1) with the energy barrier and reaction energy of 45.48 kcal/mol and −11.49 kcal/mol, respectively. The IM2 is produced by TS2 with the energy barrier and reaction energy of 52.45 kcal/mol and −5.77 kcal/mol, respectively. These two processes are similar in that an O atom from NO^+^NO_3_^−^ fragments transforms into SO_2_ to lead to the formation of SO_3_. As shown in Table S1, the reaction SO_2_-2NO_2_→IM1 is exothermic and spontaneous (ΔH of −24.50 kcal/mol and ΔG of −0.29 kcal/mol at 298.15K). Due to the absence of H_2_O molecule, the hydrolysis process cannot be carried out, and HONO cannot be generated, finally.

#### 2.1.2. The Reaction of SO_2_-2NO_2_-H_2_O

**Figure 2:**
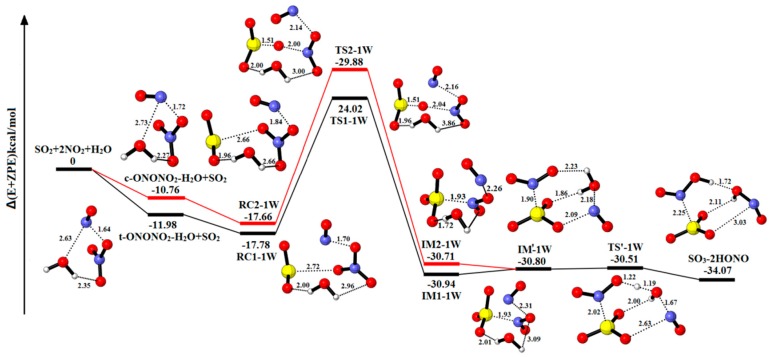
Potential energy profile for the reaction of SO_2_-2NO_2_-H_2_O. The yellow spheres are S atoms, the red spheres are O atom, the blue spheres are N atom, and the white spheres represent H atom, respectively. (Unit: Energies in kcal/mol; bond lengths in angstroms). The black and red lines are the different pathways. The black line represents the reaction of t-ONONO_2_-H_2_O-SO_2_, and the red line is the reaction of c-ONONO_2_-H_2_O-SO_2_.

Figure 2 explores the equilibrium structures and potential energy of SO_2_-2NO_2_-H_2_O. The corresponding energy data and the numerical value of charge distribution are put in Table 1 and Table 2. The relative energies, enthalpies and Gibbs free energies in all relevant species for the SO_2_-2NO_2_-H_2_O are listed in Table S2. In these two pathways, the complexes (t-ONONO_2_-H_2_O and c-ONONO_2_-H_2_O) are firstly produced via the interaction between an N_2_O_4_ molecule and a water molecule. Through a collision with SO_2_, the two complexes can transform into the pre-reactive complex (RC1-1W and RC2-1W).

Beginning with the reactant complex RC1-1W (a binding energy of 17.78 kcal/mol), the complex IM1-1W (a binding energy 30.94 kcal/mol) can be formed via the TS1-1W with the energy barrier of 41.80 kcal/mol. In this process, an oxygen atom from NO^+^NO_3_^−^ fragment is transferred to SO_2_, leading to the formation of SO_3_. The complex IM1-1W can undergo an isomerization process to form IM′-1W with the energy absorption of 0.14 kcal/mol. Because the H_2_O molecule gets involved, the NO^+^NO_2_^−^ fragments are divided into two parts, and the NO^+^ fragment rotates by an angle. Once the IM′-1W is produced, the final complex SO_3_-2HONO can be easily formed with the energy barrier of 0.29 kcal/mol and the reaction energy of −3.27 kcal/mol. From IM′-1W to SO_3_-2HONO, the water molecule reacts with the isolated NO^+^ fragment and NO_2_^−^ fragment to form two HONO molecules. In the other pathway, the reaction process is similar in the above step. The IM2-1W is formed from the conversion of RC2-1W via the transition state TS2-1W with the energy barrier of 47.54 kcal/mol and the reaction energy of −13.05 kcal/mol. After that, the hydrolysis process is the same as the above reaction (IM′-1W to SO_3_-2HONO). The reactant (IM′-1W) is formed via IM2-1W releasing the energy of 0.09 kcal/mol. In the same way, the SO_3_-2HONO is formed with the energy barrier and reaction energy of 0.30 kcal/mol and −3.27 kcal/mol, respectively.

In the absence of water, the energy barrier is higher than one H_2_O molecule and the hydrolysis reaction cannot produce HONO. The result indicates that H_2_O molecule reacts as the solvent to reduce activation energy and stable structures and also is a critical reactant in the formation of HONO.

#### 2.1.3. The Reaction of SO_2_-2NO_2_-2H_2_O

**Figure 3:**
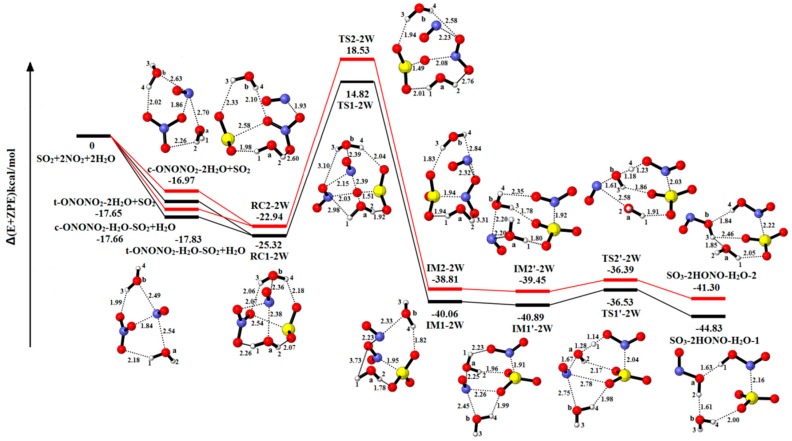
Potential energy profile for the reaction of SO_2_-2NO_2_-2H_2_O. The yellow spheres are S atoms, the red spheres are O atom, the blue spheres are N atom, and the white spheres represent H atom, respectively. (Unit: Energies in kcal/mol; bond lengths in angstroms). The black and red lines are the different pathways. The black line represents the reaction of t-ONONO_2_-2H_2_O-SO_2_, and the red line is the reaction of c-ONONO_2_-2H_2_O-SO_2_.

Figure 3 presents the optimized geometries of molecular species involved in the reaction of SO_2_-2NO_2_-2H_2_O to examine the role of an additional H_2_O molecule. Table 1 and Table 2 show the corresponding energy data and the charge distribution. Table S3 shows the relative energies, enthalpies and Gibbs free energies in all relevant species for the SO_2_-2NO_2_-2H_2_O. In these pathways, the pre-reactive complexes (RC1-2W and RC2-2W) are produced via the interaction between the complexes (t-ONONO_2_-2H_2_O and c-ONONO_2_-2H_2_O) and SO_2_, or a collision of the complexes (t-ONONO_2_-H_2_O-SO_2_ and c-ONONO_2_-H_2_O-SO_2_) with H_2_O.

Starting with the complex RC1-2W (−25.32 kcal/mol), the intermediate complex IM1-2W (−40.06 kcal/mol) is formed via TS1-2W with the energy barrier of 40.14 kcal/mol. The isomerized intermediate complex IM1′-2W (−40.89 kcal/mol) is formed by the H_2_O_(a)_ closing to NO^+^NO_2_^−^. The H_2_O_(a)_ molecule reacts with NO^+^NO_2_^−^ with an energy barrier and reaction energy of 4.36 kcal/mol and −3.94 kcal/mol, respectively. The other pathway is analogous with the above process, and the barrier (41.47 kcal/mol) is slightly higher than the reaction pathway involving in TS1-2W.

In the presence of two H_2_O molecules, the H_2_O_(a)_ serves as reactant to form HONO, and the other H_2_O_(b)_ acts as a solvent molecule in this reaction pathway to reduce energy barrier and stable complex structures. Compare with the one-water reaction in the first step, the energy barrier for TS1-2W is reduced by 1.66 kcal/mol. Moreover, the energy barrier of TS2-2W is decreased by 6.07 kcal/mol for TS2-1W. It shows that the second-water molecule plays a certain role in lowering the energy barrier and stabilizing the complexes structures.

#### 2.1.4. The Reaction of SO_2_-2NO_2_-3H_2_O

**Figure 4:**
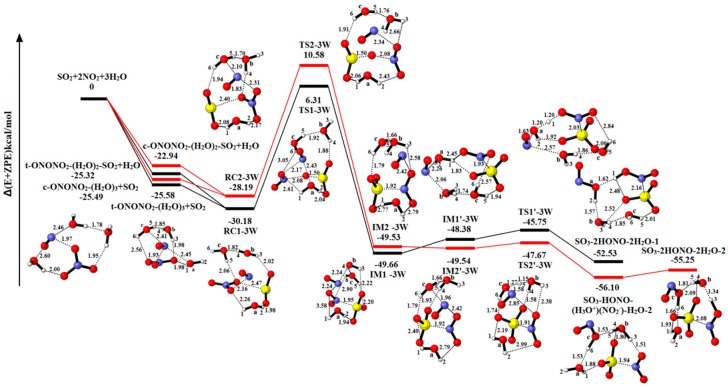
Potential energy profile for the reaction of SO_2_-2NO_2_-3H_2_O. The yellow spheres are S atoms, the red spheres are O atom, the blue spheres are N atom, and the white spheres represent H atom, respectively. (Unit: Energies in kcal/mol; bond lengths in angstroms). The black and red lines are the different pathways. The black line represents the reaction of t-ONONO_2_-3H_2_O-SO_2_, and the red line is the reaction of c-ONONO_2_-3H_2_O-SO_2_.

The consequence of three H_2_O molecules taking part in reaction is shown in Figure 4. The corresponding energy data and the charge distribution are put in Table 1 and Table 2. Table S4 summarizes the relative energies, enthalpies and Gibbs free energies in all relevant species for the SO_2_-2NO_2_-3H_2_O. The reactant complex RC1-3W (−30.18 kcal/mol) and RC2-3W (−28.19 kcal/mol) may be formed after SO_2_ attacks the tetramer complexes (t-ONONO_2_-(H_2_O)_3_ and c-ONONO_2_-(H_2_O)_3_), or H_2_O attacks t-ONONO_2_-(H_2_O)_2_-SO_2_ and t-ONONO_2_-(H_2_O)_2_-SO_2_, respectively. To form the IM1-3W from RC1-3W, there is an energy barrier of 36.49 kcal/mol at the transition state TS1-3W and reducing reaction energy of 19.48 kcal/mol. The reactant complex of hydrolysis reaction is IM1′-3W, and NO^+^NO_2_^−^ is divided by the closing of H_2_O molecule. Through the TS1’-3W (−45.75 kcal/mol), a stable product complex SO_3_-2HONO-2H_2_O-1 (−52.53 kcal/mol) is produced eventually. The oxidation reaction of the other pathway is approximately similar to RC1-3W to IM1-3W. It is different from the above hydrolytic processes, and H_2_O_(c)_ reacts as a transporter to transmit a proton forming H_3_O^+^ intermediate. The relevant energy barrier and reaction energy of the process are shown in Table 1. As shown in Table S4, the reaction SO_2_-2NO_2_-3H_2_O→SO_3_-2HONO-2H_2_O-1 is exothermic and spontaneous.

In comparison to one-water molecules in the process of SO_2_ oxidation, three H_2_O molecules have successfully reduced the energy barrier height by 5.35 kcal/mol and 8.77 kcal/mol. In these two pathways, the two additional H_2_O molecules act only as a solvent without entering into the reaction. It shows that the third-water molecule plays a significant role in stabilizing the complexes and lowering the energy barrier. As the number of H_2_O increases, the releasing energy gets more and more, and the configurations of product complexes are more and more stable. The hydrolysis reaction is still favorable thermodynamically with the number of H_2_O molecule increasing.

### 2.2. The Reaction of SO_2_-2NO_2_-nH_2_O-mNH_3_ (n = 0, 1, 2; m = 1, 2)

#### 2.2.1. The Reaction of SO_2_-2NO_2_-NH_3_

**Figure 5:**
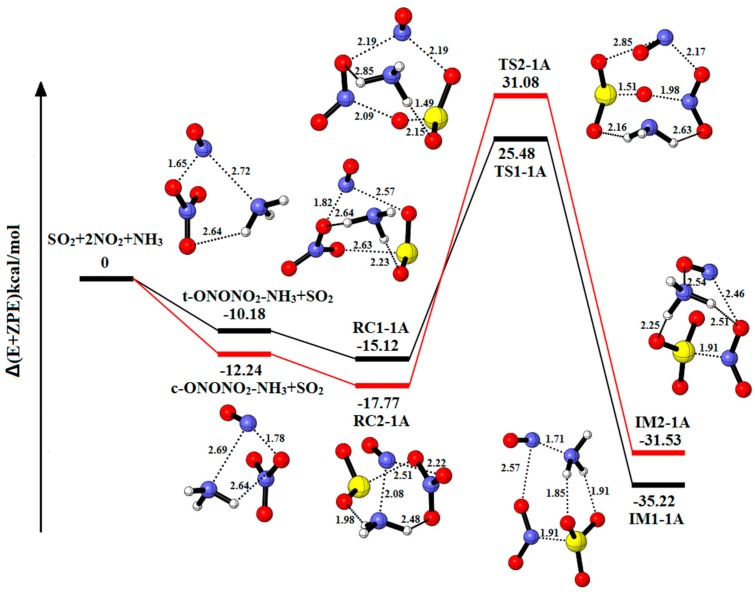
Potential energy profile for the reaction of SO_2_-2NO_2_-NH_3_. The yellow spheres are S atoms, the red spheres are O atom, the blue spheres are N atom, and the white spheres represent H atom, respectively. (Unit: Energies in kcal/mol; bond lengths in angstroms). The black and red lines are the different pathways. The black line represents the reaction of t-ONONO_2_-NH_3_-SO_2_, and the red line is the reaction of c-ONONO_2_-NH_3_-SO_2_.

Figure 5 explores the equilibrium structures and potential energy of the system 2NO_2_-SO_2_-NH_3_. Table S5 summarizes the relative energies, enthalpies and Gibbs free energies in all relevant species. The corresponding thermodynamic data and the charge distribution are put in Table 1 and Table 2. Reactant complexes (RC1-1A and RC2-1A) are formed through the collision between complexes (t-ONONO_2_-NH_3_ and c-ONONO_2_-NH_3_) and SO_2_, which is equivalent to replacing one H_2_O molecule from RC1-1W and RC2-1W with NH_3_. RC1-1A (−15.12 kcal/mol) is converted into IM1-1A (−35.22 kcal/mol) via the transformation of an O molecule with the energy barrier and reaction energy of 40.60 kcal/mol and −20.10 kcal/mol, respectively. The other pathway is similar to the above pathway with the energy barrier and reaction energy of 48.85 kcal/mol and −13.76 kcal/mol, respectively. The hydrolytic process cannot occur because of the absence of H_2_O molecule. As we can see in Table S5, the reaction SO_2_-2NO_2_-NH_3_→IM1-1A is exothermic and spontaneous (ΔH = −37.16 kcal/mol and ΔG = −3.12 kcal/mol).

Compared with the reaction pathway of SO_2_-2NO_2_ to IM1 and IM2, the result indicates that NH_3_ molecule can serve as catalyst and solvent to reduce the energy barrier and stable structures. The NH_3_ molecule cannot trigger the hydrolysis reaction to produce HONO.

#### 2.2.2. The Reaction of SO_2_-2NO_2_-H_2_O-NH_3_

**Figure 6:**
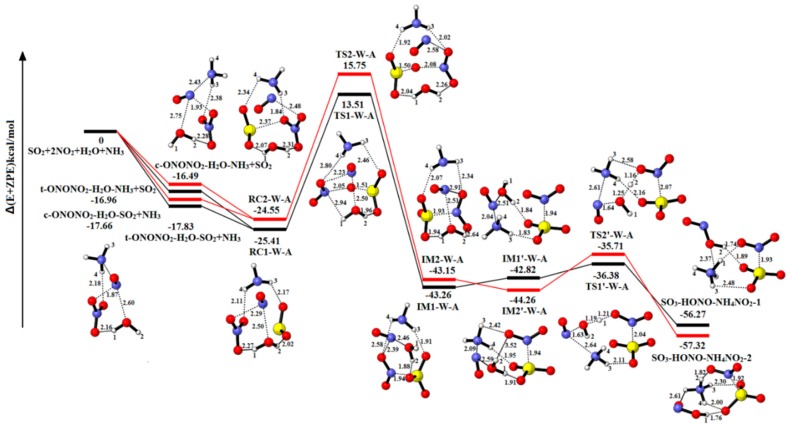
Potential energy profile for the reaction of SO_2_-2NO_2_-H_2_O-NH_3_. The yellow spheres are S atoms, the red spheres are O atom, the blue spheres are N atom, and the white spheres represent H atom, respectively. (Unit: Energies in kcal/mol; bond lengths in angstroms). The black and red lines are the different pathways. The black line represents the reaction of t-ONONO_2_-H_2_O-NH_3_-SO_2_, and the red line is the reaction of c-ONONO_2_-H_2_O-NH_3_-SO_2_.

The relative energies, enthalpies and Gibbs free energies in all relevant species for SO_2_-2NO_2_-H_2_O-NH_3_ are summarized in Table S6. As shown in Figure 6, RC1-1W-1A and RC2-1W-1A are formed after SO_2_ attacks the complex t/c-ONONO_2_-H_2_O-NH_3_ or NH_3_ attacks t/c-ONONO_2_-H_2_O-SO_2_. The product complex IM1-W-A (−43.26 kcal/mol) is formed through TS1-W-A (13.51 kcal/mol) with the energy barrier and reaction energy of 38.92 kcal/mol and −17.85 kcal/mol, respectively. When the only water molecule gets close to the NO^+^NO_2_^−^, the reactant complex (IM1′-W-A) of hydrolysis reaction is formed through separating the two parts, NO^+^ and NO_2_^−^. The IM1′-W-A releases the reaction energy of 13.45 kcal/mol to form the product complex SO_3_-HONO-NH_4_NO_2_-1 (−56.27 kcal/mol) via TS1’-W-A with the energy barrier of 6.44 kcal/mol. The NH_3_ molecule and the H_2_O molecule not only serve as catalyst and stabilizer, but also act as reactants to produce HONO and NH_4_NO_2_. It is different from the pathway of two H_2_O molecules, because the NH_3_ is more protophilia than water and the HONO is liable to provide a proton to form NH_4_NO_2_. The other pathway is analogous with RC1-W-A to SO_3_-HONO-NH_4_NO_2_-1. The energy barrier and energy reaction are 40.30 kcal/mol and −18.60 kcal/mol in oxidation reaction and 8.55 kcal/mol and −13.06 kcal/mol in hydrolysis reaction, respectively.

Compared with the one-water reaction, the energy barrier for the SO_2_-oxidation reaction is reduced by 2.92 kcal/mol and 7.24 kcal/mol, respectively. Compared with the two-water reaction, it shows that the NH_3_ molecule plays a more important role in stabilizing the complexes and lowering the energy barrier. When ammonia (NH_3_) is present in hydrolysis reaction, the product complexes form ammonium nitrite (NH_4_NO_2_). The most important reason is that the ability of NH_3_ to acquire the proton is stronger than water.

#### 2.2.3. The Reaction of SO_2_-2NO_2_-2H_2_O-NH_3_

**Figure 7:**
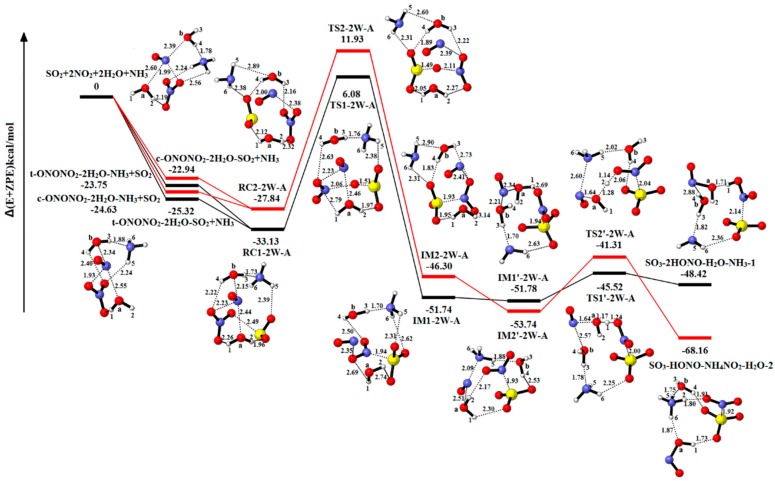
Potential energy profile for the reaction of SO_2_-2NO_2_-2H_2_O-NH_3_. The yellow spheres are S atoms, the red spheres are O atom, the blue spheres are N atom, and the white spheres represent H atom, respectively. (Unit: Energies in kcal/mol; bond lengths in angstroms). The black and red lines are the different pathways. The black line represents the reaction of t-ONONO_2_-2H_2_O-NH_3_-SO_2_, and the red line is the reaction of c-ONONO_2_-2H_2_O-NH_3_-SO_2_.

The structure of reactant complex RC1-2W-A and RC2-2W-A is formed by replacing one H_2_O molecule from RC1-3W and RC2-3W with NH_3_ in Figure 7. IM1-2W-A (−51.74 kcal/mol) is formed via TS1-2W-A (6.08 kcal/mol), in which the energy barrier and energy reaction are 39.21 kcal/mol and −18.61 kcal/mol, respectively. IM1′-2W-A (−51.78 kcal/mol) is formed by the migration process of H_2_O_(a)_ molecule. The IM1′-2W-A is converted into the product complex SO_3_-2HONO-H_2_O-NH_3_-1 (−48.42 kcal/mol) via the transition state TS1’-2W-A (−45.52 kcal/mol). The corresponding reaction energy and energy barrier are 3.36 kcal/mol and 6.26 kcal/mol, respectively. On account of the similarity to the above pathway, we will not describe the details about the other pathway. The energy barrier and reaction energy are shown in the Table 1. As shown in Table S7, the reaction SO_2_-2NO_2_-2H_2_O-NH_3_→SO_3_-2HONO-H_2_O-NH_3_-2 is exothermic and spontaneous.

In presence of one NH_3_ molecule, the NH_3_ molecule and two H_2_O molecules serve as solvent to reduce energy barrier and stable structures, and the one H_2_O molecule and the NH_3_ molecule serve as reactant to produce HONO and NH_4_NO_2_.

#### 2.2.4. The Reaction of SO_2_-2NO_2_-H_2_O-2NH_3_

**Figure 8:**
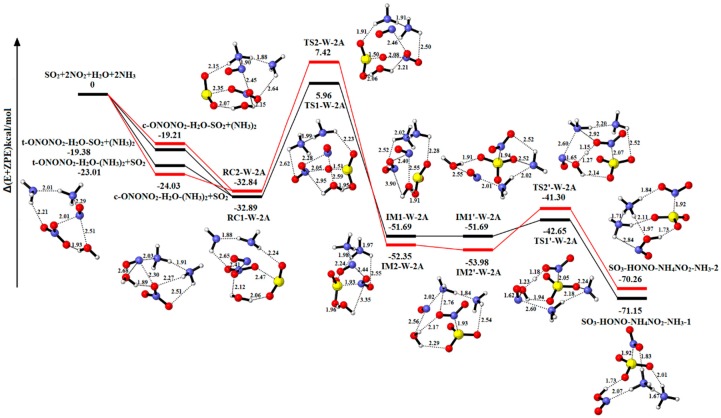
Potential energy profile for the reaction of SO_2_-2NO_2_-H_2_O-2NH_3_. The yellow spheres are S atoms, the red spheres are O atom, the blue spheres are N atom, and the white spheres represent H atom, respectively. (Unit: Energies in kcal/mol; bond lengths in angstroms). The black and red lines are the different pathways. The black line represents the reaction of t-ONONO_2_-H_2_O-2NH_3_-SO_2_, and the red line is the reaction of c-ONONO_2_-H_2_O-2NH_3_-SO_2_.

Figure 8 explores the equilibrium structures and potential energy of SO_2_-2NO_2_-H_2_O-2NH_3_. Table S8 summarizes the relative energies, enthalpies and Gibbs free energies in all relevant species. Similar to the previous reactions, the reactant complexes (RC1-W-2A and RC2-W-2A) are formed through combining NH_3_-NH_3_ and t/c-ONONO_2_-H_2_O-SO_2_ or SO_2_ and t/c-ONONO_2_-H_2_O-(NH_3_)_2_, which equals replacing two H_2_O molecules from RC1-3W or RC2-3W with two NH_3_ molecules. Starting with the reactant complex RC1-W-2A, the reaction occurs through the transition state TS1-W-2A forming the product complex IM1-W-2A. The process requires an energy barrier of 38.85 kcal/ and reaction energy of −18.80 kcal/mol, respectively. The IM1′-W-2A, with a binding energy of 51.69 kcal/mol, can serve as the reactant complex in the next step. The SO_3_-HONO-NH_4_NO_2_-NH_3_-1 (−71.15 kcal/mol) is resulted from IM1′-W-2A by the only H_2_O molecule reacting between the NO^−^ and NO_2_^−^, along with energy barrier and energy reaction of 9.04 kcal/mol and −19.46 kcal/mol, respectively. As shown in Figure 8, the other pathway is similar to the reaction of RC1-W-2A to SO_3_-HONO-NH_4_NO_2_-NH_3_-1. There are energy barrier and reaction energy of 40.26 kcal/mol and −19.51 kcal/mol in oxidation reaction, respectively. In the hydrolysis process, the energy barrier and reaction energy are 12.68 kcal/mol and −16.28 kcal/mol, respectively. Table S8 indicates that both reaction pathways SO_2_-2NO_2_-H_2_O-2NH_3_→SO_3_-HONO-NH_4_NO_2_-1 and SO_3_-HONO-NH_4_NO_2_-2 are exothermic and spontaneous.

In presence of two NH_3_ molecules, two NH_3_ molecules and the only H_2_O molecule serve as catalyst and stabilizer, and the one NH_3_ molecule and H_2_O molecule serve as reactant to produce NH_4_NO_2_.

Compared with the three-water reaction, the proportion of H_2_O and NH_3_ molecules is 2:1 and 1:2. The result indicates that the energy barrier is slighting different from the three-water molecule in the SO_2_-oxidation reaction. In the hydrolytic reaction, the energy barrier is higher than the three-water molecule, and these processes release more reaction energy. Moreover, the binding energy of product complexes is lower than that of the SO_3_-2HONO-2H_2_O. This result shows that the NH_3_ molecule plays a significant role in stabilizing complexes.

## 3. Materials and Methods 

All quantum chemistry calculations are performed by Gaussian 09 programs (vB.01, Gaussian, Inc, Wallingford, CT, USA) [36]. The structures of the reactant (RC), product (PC), intermediate (IM), and transition states (TS) are optimized using M06-2X density functional method with the 6-311++G (d, p) basis set [37]. This is relatively accurate and time-efficient when used to study the reaction mechanism and has been employed successfully in our previous studies [38,39]. The vibrational frequencies have been obtained to verify that the reactant complexes, intermediates, and product complexes have all positive frequencies and that the transition state (TS) geometries have only one imaginary frequency at the same level. According to the calculation of vibrational frequencies, zero-point energy (ZPE) and thermal correction are also obtained at the same level to decide their characteristics and thermodynamic properties. All the enthalpies (H) and Gibbs free energies (G) are calculated with thermal correction at 298.15 K. Moreover, the intrinsic reaction coordinate (IRC) calculation is used to verify whether each transition state is connected with the corresponding intermediates [40]. The single-point energies of all stationary points are refined using the more precise basis set for M06-2X/6-311++G (3df, 3pd) [41]. The ultrafine integration grid is applied to improving the accuracy during the whole gas-phase calculation. We also calculated electrostatic potential (ESP) to analyze charge distribution for NO^+^NO_3_^−^ and NO^+^NO_2_^−^ fragments of RC and IM with the same basis [42,43]. The geometries are visualized using the CYL view software package (v1.0b, Université de Sherbrooke, Montreal, QC, Canada) [44].

## 4. Conclusions

In this paper, the reaction mechanism of SO_2_ with NO_2_ to form SO_3_ in the presence of H_2_O and NH_3_ is investigated. Table S9 represents that Cartesian coordinates for all relative Optimized geometries (reactants, transient states and products) at M06-2X/6-311++G(d,p), x coordinate, y coordinate and z coordinate. The effects of different amounts of H_2_O and NH_3_ have been studied in detail. The results indicate that HONO cannot be formed in the absence of H_2_O molecules. In the oxidation step of the system SO_2_-2NO_2_-nH_2_O (*n* = 0, 1, 2, 3), H_2_O plays a catalytic role to produce SO_3_, which reduces the energy barrier. In the hydrolytic step, the energy barrier of two H_2_O molecules is larger than that of one or three H_2_O molecules. But it is still thermodynamically favorable. When NH_3_ is involved in the reaction, the energy barrier is lower than SO_2_-2NO_2_ in the oxidation step. For SO_2_-2NO_2_-2H_2_O, when the ratio of NH_3_ to H_2_O is 1:1, the energy barrier changes more greatly than SO_2_-2NO_2_-2H_2_O. For SO_2_-2NO_2_-3H_2_O, when the ratio of NH_3_ to H_2_O is 1:2 and 2:1, the change of the energy barrier is not obvious. Compared to pure water reactions, the role of NH_3_ in stabilizing product complexes and acquiring protons is more effective than H_2_O. This research provides a new insight into reaction pathways of sulfuric acid formation, and can also contribute to the further study of aerosol surface reactions.

## Figures and Tables

**Table 1:** Energy barriers and reaction energies for the integrated reactions 2NO_2_-SO_2_-mH_2_O-nNH_3_ (*m* = 0, 1, 2, 3; *n* = 0, 1, 2) (unit: kcal/mol).

	Oxidation Reaction		Hydrolysis Reaction	
Reaction Systems	Energy Barrier (kcal/mol)	Reaction Energy (kcal/mol)	Energy Barrier (kcal/mol)	Reaction Energy (kcal/mol)
SO_2_-2NO_2_	45.48	−11.49		
	52.45	−5.77		
SO_2_-2NO_2_-H_2_O	41.80	−13.16	0.29	−3.27
	47.54	−13.05	0.29	−3.27
SO_2_-2NO_2_-2H_2_O	40.14	−14.75	4.36	−3.94
	41.47	−15.87	3.06	−1.85
SO_2_-2NO_2_-3H_2_O	36.49	−19.48	2.63	−4.15
	38.77	−21.34	1.87	−6.56
SO_2_-2NO_2_-NH_3_	40.60	−20.10		
	48.85	−13.76		
SO_2_-2NO_2_-H_2_O-NH_3_	38.92	−17.85	6.44	−13.45
	40.30	−18.60	8.55	−13.06
SO_2_-2NO_2_-2H_2_O-NH_3_	39.21	−18.61	6.26	3.36
	39.77	−18.46	12.43	−14.42
SO_2_-2NO_2_-H_2_O-2NH_3_	38.85	−18.80	9.04	−19.46
	40.26	−19.51	12.68	−16.28

**Table 2:** The charge distribution for the NO^+^NO_3_^−^ and NO^+^NO_2_^−^ in reactant complexes (RC) and intermediates (IM).

	Reactant Complexes	NO^+^	NO_3_^−^	Intermediate	NO^+^	NO_2_^−^
SO_2_-2NO_2_	RC1	0.330	−0.328	IM1	0.502	−0.201
	RC2	0.405	−0.394	IM2	0.501	−0.217
SO_2_-2NO_2_-H_2_O	RC1-1W	0.472	−0.437	IM1-1W	0.712	−0.250
	RC2-1W	0.506	−0.472	IM2-1W	0.688	−0.280
SO_2_-2NO_2_-2H_2_O	RC1-2W	0.644	−0.605	IM1-2W	0.772	−0.290
	RC2-2W	0.592	−0.532	IM2-2W	0.726	−0.259
SO_2_-2NO_2_-3H_2_O	RC1-3W	0.730	−0.695	IM1-3W	0.767	−0.309
	RC2-3W	0.705	−0.724	IM2-3W	0.621	−0.282
SO_2_-2NO_2_-NH_3_	RC1-1A	0.448	−0.442	IM1-1A	0.241	−0.222
	RC2-1A	0.458	−0.635	IM2-1A	0.643	−0.228
SO_2_-2NO_2_-H_2_O-NH_3_	RC1-1W-1A	0.599	−0.569	IM1-1W-1A	0.640	−0.261
	RC2-1W-1A	0.508	−0.672	IM2-1W-1A	0.596	−0.279
SO_2_-2NO_2_-2H_2_O-NH_3_	RC1-2W-1A	0.675	−0.673	IM1-2W-1A	0.773	−0.296
	RC2-2W-1A	0.722	−0.625	IM2-2W-1A	0.717	−0.241
SO_2_-2NO_2_-H_2_O-2NH_3_	RC1-1W-2A	0.440	−0.696	IM1-1W-2A	0.549	−0.297
	RC2-1W-2A	0.449	−0.691	IM2-1W-2A	0.488	−0.283

## Supplementary Materials

The following are available online at https://www.mdpi.com/1422-0067/20/15/3746/s1.

## Abbreviations

PM	Particulate Matter
DFT	Density Functional Theory
RC	Reactant complex
IM	Intermediate
PC	Product
TS	Transient state
IRC	Intrinsic reaction coordinate
ESP	Electrostatic potential

## References

[1] You C.F., Xu X.C. (2010). Coal combustion and its pollution control in China. Energy.

[2] Hewitt C.N. (2001). The atmospheric chemistry of sulphur and nitrogen in power station plumes. Atmos. Environ..

[3] Rosas J.M., Ruiz-Rosas R., Rodríguez-Mirasol J., Cordero T. (2017). Kinetic study of SO_2_ removal over lignin-based activated carbon. Chem. Eng. J..

[4] Baltrusaitis J., Jayaweera P.M., Grassian V.H. (2011). Sulfur Dioxide Adsorption on TiO_2_ Nanoparticles: Influence of Particle Size, Coadsorbates, Sample Pretreatment, and Light on Surface Speciation and Surface Coverage. J. Phys. Chem. C.

[5] Kroll J.A., Frandsen B.N., Kjaergaard H.G., Vaida V. (2018). Atmospheric Hydroxyl Radical Source: Reaction of Triplet SO_2_ and Water. J. Phys. Chem. A.

[6] Wang H., You C. (2016). Photocatalytic removal of low concentration SO_2_ by titanium dioxide. Chem. Eng. J..

[7] Zhang R., Wang G., Guo S., Zamora M.L., Ying Q., Lin Y., Wang W., Hu M., Wang Y. (2015). Formation of urban fine particulate matter. Chem Rev..

[8] Hung H.M., Hoffmann M.R. (2015). Oxidation of Gas-Phase SO_2_ on the Surfaces of Acidic Microdroplets: Implications for Sulfate and Sulfate Radical Anion Formation in the Atmospheric Liquid Phase. Environ. Sci. Technol..

[9] Long B., Bao J.L., Truhlar D.G. (2017). Reaction of SO_2_ with OH in the atmosphere. Phys. Chem. Chem. Phys. Pccp.

[10] Sitha S., Jewell L.L., Piketh S.J., Fourie G. (2011). A quantum chemical calculation of the potential energy surface in the formation of HOSO_2_ from OH + SO_2_. Atmos. Environ..

[11] Zhang R., Khalizov A., Wang L., Hu M., Xu W. (2012). Nucleation and growth of nanoparticles in the atmosphere. Chem. Rev..

[12] Charlson R.J., Schwartz S.E., Hales J.M., Cess R.D., Coakley J.A., Hansen J.E., Hofmann D.J. (1992). Climate forcing by anthropogenic aerosols. Science.

[13] Zheng G.J., Duan F.K., Su H., Ma Y.L., Cheng Y., Zheng B., Zhang Q., Huang T., Kimoto T., Chang D. (2015). Exploring the severe winter haze in Beijing: The impact of synoptic weather, regional transport and heterogeneous reactions. Atmos. Chem. Phys..

[14] Wang Y., Lampel J., Xie P., Beirle S., Li A., Wu D., Wagner T. (2017). Ground-based MAX-DOAS observations of tropospheric aerosols, NO_2_, SO_2_ and HCHO in Wuxi, China, from 2011 to 2014. Atmos. Chem. Phys..

[15] Quan J., Tie X., Zhang Q., Liu Q., Li X., Gao Y., Zhao D. (2014). Characteristics of heavy aerosol pollution during the 2012–2013 winter in Beijing, China. Atmos. Environ..

[16] Zhao X.J., Zhao P.S., Xu J., Meng W., Pu W.W., Dong F., He D., Shi Q.F. (2013). Analysis of a winter regional haze event and its formation mechanism in the North China Plain. Atmos. Chem. Phys..

[17] Xue J., Yuan Z., Yu J.Z., Lau A.K.H. (2014). An Observation-Based Model for Secondary Inorganic Aerosols. Aerosol Air Qual. Res..

[18] He H., Wang Y., Ma Q., Ma J., Chu B., Ji D., Tang G., Liu C., Zhang H., Hao J. (2014). Mineral dust and NOx promote the conversion of SO_2_ to sulfate in heavy pollution days. Sci. Rep..

[19] Cheng Y., Zheng G., Wei C., Mu Q., Zheng B., Wang Z., Gao M., Zhang Q., He K., Carmichael G. (2016). Reactive nitrogen chemistry in aerosol water as a source of sulfate during haze events in China. Sci. Adv..

[20] Wang Y., Zhang Q., Jiang J., Zhou W., Wang B., He K., Duan F., Zhang Q., Philip S., Xie Y. (2014). Enhanced sulfate formation during China’s severe winter haze episode in January 2013 missing from current models. J. Geophys. Res. Atmos..

[21] Gao M., Carmichael G.R., Wang Y., Ji D., Liu Z., Wang Z. (2016). Improving simulations of sulfate aerosols during winter haze over Northern China: The impacts of heterogeneous oxidation by NO_2_. Front. Environ. Sci. Eng..

[22] Xue J., Yuan Z., Griffith S.M., Yu X., Lau A.K.H., Yu J.Z. (2016). Sulfate Formation Enhanced by a Cocktail of High NO_x_, SO_2_, Particulate Matter, and Droplet pH during Haze-Fog Events in Megacities in China: An Observation-Based Modeling Investigation. Environ. Sci. Technol..

[23] Ma J., Chu B., Liu J., Liu Y., Zhang H., He H. (2018). NO_x_ promotion of SO_2_ conversion to sulfate: An important mechanism for the occurrence of heavy haze during winter in Beijing. Environ. Pollut..

[24] Zhang H., Chen S., Jie Z., Zhang S., Zhang Y., Zhang X., Li Z., Zeng X. (2018). Formation of aqueous-phase sulfate during the haze period in China: Kinetics and atmospheric implications. Atmos. Environ..

[25] Wang G., Zhang R., Gomez M.E., Yang L., Levy Zamora M., Hu M., Lin Y., Peng J., Guo S., Meng J. (2016). Persistent sulfate formation from London Fog to Chinese haze. Proc. Natl. Acad. Sci. United States Am..

[26] Pimentel A.S., Lima F.C.A., da Silva A.B.F. (2007). The asymmetric dimerization of nitrogen dioxide. Chem. Phys. Lett..

[27] Miller Y., Finlayson-Pitts B.J., Gerber R.B. (2009). Ionization of N_2_O_4_ in Contact with Water: Mechanism, Time Scales and Atmospheric Implications. J. Am. Chem. Soc..

[28] Liu C., Ma Q., Liu Y., Ma J., He H. (2012). Synergistic reaction between SO_2_ and NO_2_ on mineral oxides: A potential formation pathway of sulfate aerosol. Phys. Chem. Chem. Phys..

[29] Ma Q., Liu Y., He H. (2008). Synergistic Effect between NO_2_ and SO_2_ in Their Adsorption and Reaction on γ-Alumina. J. Phys. Chem. A.

[30] Ma Q., Wang T., Liu C., He H., Wang Z., Wang W., Liang Y. (2017). SO_2_ Initiates the Efficient Conversion of NO_2_ to HONO on MgO Surface. Environ. Sci. Technol..

[31] Zhu R.S., Lai K.Y., Lin M.C. (2012). Ab initio chemical kinetics for the hydrolysis of N_2_O_4_ isomers in the gas phase. J. Phys. Chem A.

[32] Liu W.G., Goddard W.A. (2012). First-principles study of the role of interconversion between NO_2_, N_2_O_4_, cis-ONO-NO_2_, and trans-ONO-NO_2_ in chemical processes. J. Am. Chem Soc..

[33] Wang X., Bai F.Y., Sun Y.Q., Wang R.S., Pan X.M., Tao F.M. (2015). Theoretical study of the gaseous hydrolysis of NO_2_ in the presence of NH_3_ as a source of atmospheric HONO. Environ. Chem..

[34] Varner M.E., Finlayson-Pitts B.J., Benny Gerber R. (2014). Reaction of a charge-separated ONONO_2_ species with water in the formation of HONO: An MP2 Molecular Dynamics study. Phys. Chem. Chem. Phys..

[35] Addison C.C. (1980). Dinitrogen tetroxide, nitric acid, and their mixtures as media for inorganic reactions. Chem. Rev..

[36] Frisch M.J., Trucks G.W., Schlegel H.B., Scuseria G.E., Robb M.A., Cheeseman J.R., Scalmani G., Barone V., Mennucci B., Petersson G.A. (2010). Gaussian 09, Revision B.01.

[37] Zhao Y., Truhlar D.G. (2008). The M06 suite of density functionals for main group thermochemistry, thermochemical kinetics, noncovalent interactions, excited states, and transition elements: Two new functionals and systematic testing of four M06-class functionals and 12 other functionals. Theor. Chem. Acc..

[38] Lv G., Nadykto A.B., Sun X., Zhang C., Xu Y. (2018). Towards understanding the role of amines in the SO_2_ hydration and the contribution of the hydrated product to new particle formation in the Earth’s atmosphere. Chemosphere.

[39] Lv G., Sun X., Zhang C., Li M. (2019). Understanding the catalytic role of oxalic acid in SO_3_ hydration to form H_2_SO_4_ in the atmosphere. Atmos. Chem. Phys..

[40] Fukui K. (1981). The Path of Chemical Reactions-The IRC Approach. Acc. Chem. Res..

[41] Purvis G.D., Bartlett R.J. (1982). A full coupled-cluster singles and doubles model: The inclusion of disconnected triples. J. Chem. Phys..

[42] Besler B.H., Merz K.M., Kollman P.A. (1990). Atomic Charges Derived from Semiempirical Methods. J. Comput. Chem..

[43] Singh C.U., Kollman P.A. (1984). An Approach to Computing Electrostatic Charges for Molecules. J. Comput. Chem..

[44] Legault C.Y. (2009). CYLview, 1.0b.

