# Supplementary material for: Synergistic Reaction of SO2 with NO2 in Presence of H2O and NH3: A Potential Source of Sulfate Aerosol
Source: Int J Mol Sci. 2019 Jul 31;20(15):3746. doi: 10.3390/ijms20153746 (PMC6696214; doi:10.3390/ijms20153746)
Supplement: Supplementary file 1 [file ijms-20-03746-s001.zip › ijms-544891-supplementary.docx]

**Supplementary Information**

Synergistic Reaction of SO_2_ with NO_2_ in Presence of H_2_O and NH_3_: A Potential Source of Sulfate Aerosol

**Zehua Wang^1^, Chenxi Zhang^2^, Guochun Lv^1^, Xiaomin Sun^1*^, Ning Wang^1^ and Zhiqiang Li^3*^**

^1^ Environment Research Institute, Shandong University, Jinan 250100, China

^2^ College of Biological and Environmental Engineering, Binzhou University, Binzhou 256600, China

^3^ Center for Optics Research and Engineering (CORE), Shandong University, Qingdao 266237, China

***** Corresponding authors: Xiaomin Sun ([sxmwch@sdu.edu.cn](mailto:sxmwch@sdu.edu.cn)); Zhiqiang Li (lzq@sdu.edu.cn)

Table S1. Zero point corrected relative energies, the relative enthalpies (at 298.15 K) and Gibbs free energies (at 298.15 K) for the reaction SO_2_-2NO_2_ (in kcal/mol).

|  | △E | △H | △G |
| --- | --- | --- | --- |
| SO_2_+2NO_2_ | 0 | 0 | 0 |
| RC1 | -11.78 | -12.60 | 9.76 |
| RC2 | -10.54 | -10.89 | 10.24 |
| TS1 | 33.70 | 33.01 | 55.40 |
| TS2 | 41.91 | 41.09 | 63.98 |
| IM1 | -23.27 | -24.50 | -0.29 |
| IM2 | -16.31 | -17.20 | 6.23 |

Table S2. Zero point corrected relative energies, the relative enthalpies (at 298.15 K) and Gibbs free energies (at 298.15 K) for the reaction SO_2_-2NO_2_-H_2_O (in kcal/mol).

|  | △E | △H | △G |
| --- | --- | --- | --- |
| SO_2_+2NO_2_+H_2_O | 0 | 0 | 0 |
| RC1-1W | -17.78 | -18.46 | 11.39 |
| RC2-1W | -17.66 | -18.36 | 11.16 |
| TS1-1W | 24.02 | 22.61 | 55.53 |
| TS2-1W | 29.88 | 28.77 | 61.15 |
| IM1-1W | -30.94 | -32.38 | -0.16 |
| IM2-1W | -30.71 | -32.60 | 1.69 |
| IM'-1W | -31.80 | -32.72 | 1.52 |
| TS'-1W | -31.51 | -33.13 | 2.63 |
| SO_3_-2HONO | -34.07 | -35.90 | -2.60 |

Table S3. Zero point corrected relative energies, the relative enthalpies (at 298.15 K) and Gibbs free energies (at 298.15 K) for the reaction SO_2_-2NO_2_-2H_2_O (in kcal/mol).

|  | △E | △H | △G |
| --- | --- | --- | --- |
| SO_2_+2NO_2_+2H_2_O | 0 | 0 | 0 |
| RC1-2W | -25.32 | -27.07 | 15.34 |
| RC2-2W | -22.94 | -24.16 | 16.32 |
| TS1-2W | 14.82 | 13.06 | 55.13 |
| TS2-2W | 18.53 | 16.90 | 58.26 |
| IM1-2W | -40.06 | -42.13 | 0.58 |
| IM2-2W | -38.81 | -41.01 | 2.51 |
| IM1'-2W | -40.89 | -43.04 | 0.40 |
| IM2'-2W | -39.45 | -41.01 | 2.10 |
| TS1'-2W | -36.53 | -39.21 | 4.76 |
| TS2'-2W | -36.39 | -39.38 | 5.02 |
| SO_3_-2HONO-H_2_O-1 | -44.83 | -47.25 | -4.59 |
| SO_3_-2HONO-H_2_O -2 | -41.30 | -43.67 | -0.69 |

Table S4. Zero point corrected relative energies, the relative enthalpies (at 298.15 K) and Gibbs free energies (at 298.15 K) for the reaction SO_2_-2NO_2_-3H_2_O (in kcal/mol).

|  | △E | △H | △G |
| --- | --- | --- | --- |
| SO_2_+2NO_2_+3H_2_O | 0 | 0 | 0 |
| RC1-3W | -30.18 | -32.37 | 18.92 |
| RC2-3W | -28.19 | -30.80 | 21.81 |
| TS1-3W | 6.31 | 3.89 | 56.64 |
| TS2-3W | 10.58 | 7.97 | 60.89 |
| IM1-3W | -49.66 | -52.26 | 1.22 |
| IM2-3W | -49.53 | -52.66 | 1.51 |
| IM1'-3W | -48.38 | -51.79 | 3.25 |
| IM2'-3W | -49.54 | -52.66 | 1.46 |
| TS1'-3W | -45.75 | -49.36 | 5.70 |
| TS2'-3W | -47.67 | -51.50 | 3.34 |
| SO_3_-2HONO-2H_2_O-1 | -52.53 | -55.41 | -2.20 |
| SO_3_-HONO-(H_3_O)^+^(NO_2_)^-^-H_2_O-2 | -56.10 | -60.13 | -5.46 |
| SO_3_-2HONO-H_2_O -2 | -55.25 | -58.75 | 5.89 |

Table S5. Zero point corrected relative energies, the relative enthalpies (at 298.15 K) and Gibbs free energies (at 298.15 K) for the reaction SO_2_-2NO_2_-NH_3_ (in kcal/mol).

|  | △E | △H | △G |
| --- | --- | --- | --- |
| SO_2_+2NO_2_+NH_3_ | 0 | 0 | 0 |
| RC1-1A | -15.12 | -15.59 | 15.11 |
| RC2-1A | -17.77 | -19.05 | 14.05 |
| TS1-1A | 25.48 | -24.65 | 56.06 |
| TS2-1A | 31.08 | 30.03 | 62.84 |
| IM1-1A | -31.53 | -37.16 | -3.12 |
| IM2-1A | -35.22 | -32.52 | 1.61 |

Table S6. Zero point corrected relative energies, the relative enthalpies (at 298.15 K) and Gibbs free energies (at 298.15 K) for the reaction SO_2_-2NO_2_- H_2_O-NH_3_ (in kcal/mol).

|  | △E | △H | △G |
| --- | --- | --- | --- |
| SO_2_+2NO_2_+H_2_O+NH_3_ | 0 | 0 | 0 |
| RC1-W-A | -25.41 | -27.07 | 15.41 |
| RC2-W-A | -24.55 | -26.27 | 16.62 |
| TS1-W-A | 13.51 | 11.72 | 54.79 |
| TS2-W-A | 15.75 | 13.96 | 56.53 |
| IM1-W-A | -43.26 | -45.30 | -2.29 |
| IM2-W-A | -43.15 | -45.39 | -1.08 |
| IM1'-W-A | -42.82 | -44.90 | -1.74 |
| IM2'-W-A | -44.26 | -46.42 | -2.45 |
| TS1'-W-A | -36.38 | -39.07 | 5.00 |
| TS2'-W-A | -35.71 | -38.48 | 6.08 |
| SO_3_-HONO-NH_4_NO_2_-1 | -56.27 | -58.96 | -15.20 |
| SO_3_-HONO-NH_4_NO_2_-2 | -57.32 | -60.28 | -15.10 |

Table S7. Zero point corrected relative energies, the relative enthalpies (at 298.15 K) and Gibbs free energies (at 298.15 K) for the reaction SO_2_-2NO_2_-2H_2_O-NH_3_ (in kcal/mol).

|  | △E | △H | △G |
| --- | --- | --- | --- |
| SO_2_+2NO_2_+2H_2_O+NH_3_ | 0 | 0 | 0 |
| RC1-2W-A | -33.13 | -35.37 | 16.37 |
| RC2-2W-A | -27.84 | -29.64 | 21.85 |
| TS1-2W-A | 6.08 | 3.77 | 56.12 |
| TS2-2W-A | 11.93 | 9.96 | 61.85 |
| IM1-2W-A | -51.74 | -54.69 | 0.00 |
| IM2-2W-A | -46.30 | -48.82 | 4.85 |
| IM1'-2W-A | -51.78 | -54.70 | -0.12 |
| IM2'-2W-A | -53.74 | -56.56 | -1.90 |
| TS1'-2W-A | -45.52 | -49.15 | 6.06 |
| TS2'-2W-A | -41.31 | -44.52 | 10.08 |
| SO_3_-2HONO-H_2_O-NH_3_-1 | -48.42 | -51.20 | 1.56 |
| SO_3_-2HONO-H_2_O-NH_3_-2 | -68.16 | -71.53 | -18.27 |

Table S8. Zero point corrected relative energies, the relative enthalpies (at 298.15 K) and Gibbs free energies (at 298.15 K) for the reaction SO_2_-2NO_2_- H_2_O-2NH_3_ (in kcal/mol).

|  | △E | △H | △G |
| --- | --- | --- | --- |
| SO_2_+2NO_2_+H_2_O+2NH_3_ | 0 | 0 | 0 |
| RC1-W-A | -32.89 | -34.78 | 16.83 |
| RC2-W-A | -32.84 | -34.89 | 17.37 |
| TS1-W-A | 5.96 | 3.73 | 56.67 |
| TS2-W-A | 7.42 | 5.11 | 58.15 |
| IM1-W-A | -51.69 | -54.27 | -0.23 |
| IM2-W-A | -52.35 | -55.06 | -0.52 |
| IM1'-W-A | -51.69 | -54.26 | -0.28 |
| IM2'-W-A | -53.98 | -56.69 | -2.02 |
| TS1'-W-A | -42.65 | -45.87 | 9.26 |
| TS2'-W-A | -41.30 | -44.40 | 10.68 |
| SO_3_-HONO-NH_4_NO_2_-1 | -71.15 | -74.78 | -20.04 |
| SO_3_-HONO-NH_4_NO_2_-2 | -70.26 | -73.75 | -18.72 |

Table S9. Cartesian coordinates for all relative Optimized geometries (reactants, transient states and products) at M06-2X/6-311++G(d,p), x coordinate, y coordinate and z coordinate.

SO_2_:

S 0.00000000 0.36838900 0.00000000

O 1.23685900 -0.36843900 0.00000000

O -1.23685900 -0.36833900 0.00000000

NO_2_:

N 0.00000000 0.00000000 0.31532900

O 0.00000000 1.09082000 -0.13795600

O 0.00000000 -1.09082000 -0.13795600

H_2_O:

O 0.00000000 0.11661700 0.00000000

H 0.76155900 -0.46647800 0.00000000

H -0.76155900 -0.46645600 0.00000000

NH_3_:

H 0.72482400 -0.60586100 0.25592400

H -0.88740100 -0.32378100 0.25609600

N -0.00008000 -0.00014000 -0.10966000

H 0.16313800 0.93061900 0.25559900

NH_3_-H_2_O:

O -1.54413700 -0.10467500 -0.00006300

H -0.58344000 0.04109400 -0.00078600

H -1.93709300 0.76900900 0.00019200

H 1.64094200 -0.63138100 -0.72969000

H 1.90801100 0.87330100 -0.15123900

N 1.37731300 0.02254900 -0.00010000

H 1.68348300 -0.37246900 0.88272400

(NH_3_)_2_:

H 2.09560700 -0.81699100 -0.30557600

H 0.67553600 0.00193300 -0.17814100

N -1.54359000 0.00013100 -0.03603700

H -2.08087100 -0.80504900 -0.33728200

H -1.50224300 -0.02361700 0.97738200

H -2.06697400 0.82731300 -0.29966800

N 1.65497500 0.00012200 0.10032700

H 2.09924900 0.81464400 -0.30674500

t-ONONO_2_:

N 1.00568500 0.02258600 -0.02696200

O 1.89911000 -0.74395100 0.14040500

O 1.01743400 1.21649500 -0.08150400

O -0.23130100 -0.60346500 -0.21234700

N -1.38237200 0.32589300 0.24415900

O -2.35564100 -0.17399800 -0.03660100

c-ONONO_2_:

N -0.84826100 0.02871400 -0.07523700

O -1.62241400 -0.55273200 0.61415500

O -0.80828400 1.17670200 -0.39609100

O 0.18647500 -0.78247100 -0.60751700

N 1.58017900 -0.37092100 -0.17039900

O 1.60379600 0.45793200 0.60438300

RC1:

N -1.34377500 -0.93866100 0.13731900

O -2.05736200 -1.81994400 -0.22709700

O -0.56987900 -0.94920600 1.07185900

O -1.39867200 0.20035700 -0.59728600

N -1.09816600 1.53705800 0.35037600

O -1.07871000 2.41424900 -0.32879500

S 1.99999300 -0.23933900 0.20219100

O 1.50424100 1.11877700 0.23462000

O 1.73709500 -1.00915300 -0.98441600

TS1:

N 1.70039700 -0.43253400 -0.10389400

O 2.53084000 -1.23961000 0.12079900

O -0.06813200 -1.20482000 -0.89104000

O 1.72366600 0.73957800 0.25821500

N 0.01512300 1.88451300 -0.38833200

O -0.09756600 2.62763600 0.39170600

S -1.28781700 -0.80568100 -0.13898600

O -1.67151300 0.61176400 -0.43060500

O -1.34274200 -1.19366800 1.25959600

IM1:

N -0.67849500 1.17578400 0.01050000

O -1.72924500 1.70479100 -0.16889200

O -0.54200500 -1.02382800 1.42623700

O 0.39221200 1.70666400 0.22699700

N 2.09984400 -0.13482300 0.33455900

O 2.87959100 0.14812500 -0.36866600

S -0.62471200 -0.72587500 0.02432100

O 0.72348500 -0.85664000 -0.67327100

O -1.71829400 -1.13820500 -0.79297600

RC2:

N -1.15393700 1.05812400 0.08442900

O -0.13663400 1.71054400 0.04807800

O -2.09303100 1.13939700 -0.66640900

O -1.22764300 0.13015000 1.05085700

N -1.60882300 -1.44600100 0.41771100

O -1.80337400 -1.45579900 -0.66470400

S 1.89946000 -0.21580100 0.31642900

O 2.89762600 0.38879800 -0.52249800

O 0.98155100 -1.14209500 -0.31755500

TS2:

N -0.52324200 1.51530700 0.06987500

O -0.40529700 1.37524200 1.24503500

O 1.02648800 0.88563900 -0.90128300

O -1.49623800 1.23435400 -0.59479900

N -1.90753300 -0.97892300 -0.43521400

O -2.35826900 -1.34389200 0.47575100

S 1.42362500 -0.45445400 -0.31538400

O 0.15881500 -1.23420400 -0.07142000

O 2.35418000 -0.47756900 0.79715700

IM2:

N -0.45586900 0.86938400 0.43849700

O 1.85935200 -0.50575100 1.19993400

O -0.95756200 1.67383700 -0.27696100

O -1.22451000 0.20276400 1.22809400

N -2.31680500 -0.81573700 0.19111500

O -2.29993700 -0.50932400 -0.85627200

S 1.33173200 -0.23104600 -0.10045600

O 1.93565000 0.75992100 -0.93700900

O 0.44963400 -1.20629500 -0.70778500

t-ONONO_2_-H_2_O:

N 1.10660400 -0.35015500 -0.07253400

O 1.82746300 0.49006000 -0.54295800

O -0.05735700 -0.55027200 -0.75250000

N -1.27697900 -0.59342200 0.34859600

O -2.23406000 -0.63792800 -0.21788000

O -0.62439300 1.95327300 0.29519600

H 0.07885100 2.05005800 -0.35671000

H -0.49203500 2.66311400 0.92744000

O 1.28907300 -1.01865100 0.90524800

c-ONONO_2_-H_2_O:

N -1.04950900 -0.23019500 0.04297900

O -1.73947600 0.75764600 0.07648000

O -1.04345600 -1.08567200 -0.80826300

O -0.18573900 -0.38202400 1.06150300

N 1.42386500 -0.72687700 0.54856600

O 1.53214700 -0.87469400 -0.53742400

O 0.95529400 1.84543000 -0.22866200

H 0.11928400 2.05648600 0.20296100

H 1.11006800 2.55752800 -0.85286700

RC1-1W:

N 1.52422300 -0.83063900 -0.56453800

O 2.33961900 -1.62157200 -0.19004800

O -1.88455100 -0.60958000 1.11797100

O 1.92066200 0.44643500 -0.62029900

N 0.62672400 1.42857300 -0.11225000

O 1.03065300 2.44761500 0.02791300

O 0.74555500 0.03157600 1.98985700

H 1.33248400 -0.60435700 2.40636200

H -0.14794300 -0.32473900 2.06481500

S -2.19407300 -0.45563600 -0.28674500

O -2.17693600 0.88518700 -0.81345300

O 0.38299900 -1.07544500 -0.90515900

TS1-1W:

N 1.73604500 -0.78326300 -0.08777300

O 2.41375600 -1.71253700 0.18383100

O -1.79264100 -0.83663200 1.12535100

O 2.09532400 0.37923300 -0.16671700

N 0.27266700 1.48384400 -0.52469500

O 0.43914400 2.53899100 -0.68278800

O -0.09019600 1.29063200 1.78604600

H 0.64861100 0.98024100 2.31807200

H -0.80379500 0.64733000 1.92806900

S -1.47141600 -0.79850200 -0.30530800

O -1.72941700 0.50268100 -0.96091500

O -0.13136300 -1.38181900 -0.66905100

IM1-1W:

N 0.68420600 1.21365200 -0.14330300

O 1.55318200 2.01752200 -0.04504900

O 0.28993800 -0.88006100 1.31235900

O -0.50010800 1.46188000 -0.36210800

N -1.74410500 -0.46377600 -0.60660500

O -2.36552300 -0.20639300 -1.44041300

O -2.42954300 0.07794700 1.57515000

H -2.50519000 0.96694300 1.93237200

H -1.54606000 -0.23439100 1.82780100

S 1.03999300 -0.67058000 0.08464300

O 0.33967600 -1.12951100 -1.12550100

O 2.46620800 -0.74793400 0.10242500

RC2-1W:

N 1.15619400 -0.88139200 -0.66572000

O 1.06500000 -1.99897500 -0.23513600

O 0.25465200 -0.26833300 -1.23689900

O 2.28520600 -0.24031100 -0.50649900

N 2.01846200 1.48416900 0.07899800

O 1.00818300 1.85640300 -0.07869400

O 0.84308500 0.02715300 1.99308200

H 1.32078600 -0.79686800 2.12580600

H -0.09107100 -0.21923500 2.00090800

S -2.21279200 -0.19136100 -0.24806000

O -2.19476400 1.22554100 -0.50450200

O -1.76731500 -0.61917300 1.06231100

TS2-1W:

N 1.42033000 -0.96554500 -0.35356800

O 1.85442600 -1.94408300 0.14675200

O -0.45491200 -1.11644200 -1.03032500

O 2.03912900 0.06432100 -0.57133400

N 1.18620700 2.03021000 -0.56285100

O 0.41755300 2.15710400 0.17358500

O 0.43332300 0.35219200 1.96578400

H 1.03067800 -0.21873300 2.45648200

H -0.44311200 -0.05329100 2.05017700

S -1.59805900 -0.36477300 -0.40179400

O -1.50203400 1.09425400 -0.60378400

O -1.94553400 -0.77537800 0.96144400

IM2-1W:

N 0.81314800 1.19441700 0.18299700

O 1.78211100 1.84303800 0.41130500

O 2.37176500 -0.86361500 -0.39059400

O -0.33813500 1.63408000 0.17157000

N -2.32092700 0.66785400 -0.38777400

O -2.25316400 0.04785400 -1.25542000

O -2.20545300 -0.72291700 1.26739100

H -2.37432800 -0.31392600 2.12216900

H -1.26532500 -1.02219000 1.29870500

S 0.97175200 -0.70418300 -0.15111400

O 0.04181200 -0.79590100 -1.26697700

O 0.43182300 -1.19664300 1.11652400

IM'-1W:

N -2.11604300 0.16579200 -0.80764400

O -2.89663300 -0.57342600 -0.86253500

O 0.33281600 -0.60895300 1.55030900

O 0.27285100 1.81593000 -0.19088200

N 1.20053100 1.03491900 -0.28302600

O 2.31668500 1.28213700 -0.61741100

O -2.00509100 0.80240300 1.27188200

H -1.33296900 0.21350800 1.66671400

H -1.50688600 1.61994500 1.13495900

S 0.76886600 -0.76402200 0.17088200

O -0.36357800 -0.97392000 -0.77100300

O 1.96127400 -1.49593100 -0.11799600

TS'-1W:

N -2.52554300 0.31361000 -0.60521500

O -3.06262300 -0.61046800 -0.30886300

O 0.18619600 -0.80429700 1.41257000

O 0.36692900 1.91566000 0.01482700

N 1.26904100 1.03785800 -0.13902900

O 2.36901200 1.34155900 -0.42094300

O -1.74473000 0.99088800 0.70819300

H -1.49668000 0.27161500 1.32541000

H -0.71080700 1.45275400 0.34854600

S 0.74175300 -0.89673900 0.07242400

O -0.23949300 -0.92859600 -0.99402000

O 2.01657900 -1.50935000 -0.11464200

SO_3_-2HONO:

N -2.73353300 0.52500900 -0.43010200

O -3.15476100 -0.54261000 -0.27893300

O 0.13375200 -0.91750200 1.35154200

O 0.52022500 2.09985300 0.01147700

N 1.38642600 1.10074100 -0.08862700

O 2.49496400 1.40969500 -0.26477300

O -1.83610500 0.90420200 0.59302100

H -1.68443100 0.13294700 1.17909300

H -0.37113900 1.69903800 0.17086900

S 0.71217000 -1.04216700 0.03595000

O -0.17325400 -0.94632500 -1.08942200

O 2.02650500 -1.57450900 -0.10966700

t-ONONO_2_-(H_2_O)_2_:

N 0.56018600 -1.15925300 0.16069400

O 1.74981300 -1.38595800 0.16552400

O -0.26742400 -1.77548900 -0.48621100

O 0.13307000 -0.15677100 0.90078700

N -0.55605100 1.10499000 -0.24337900

O -0.82759400 1.96659900 0.36378400

O 1.94702000 1.50537100 -0.45055500

H 2.25447500 0.73070800 0.03846500

H 2.58791100 1.63916300 -1.15220600

O -2.65969500 -0.22442100 -0.16485600

H -3.39936000 -0.43027100 -0.73978900

H -2.07349700 -0.99440300 -0.19546900

c-ONONO_2_-(H_2_O)_2_:

N -0.46807200 -1.08705700 0.04922900

O -1.58383100 -1.55024800 0.01483100

O 0.40086200 -1.28667800 -0.79017100

O -0.16603700 -0.28909700 1.04421200

N 0.51988000 1.32892100 0.43289500

O 0.58487300 1.38001400 -0.64993100

O 2.78224100 0.04267700 0.06984600

H 3.57243000 -0.30962600 0.48243000

H 2.24739100 -0.71873500 -0.19359700

O -2.13049900 1.35098800 -0.10843700

H -2.39668000 0.53154700 0.32630700

H -2.88667500 1.62252100 -0.63281800

RC1-2W:

N 0.62787100 -0.79907800 1.24276900

O 0.65910200 -1.99665600 1.04374600

O -0.42792400 -0.20402500 1.53918400

O 1.68499300 -0.10123600 1.10955700

N 1.39914500 0.91831400 -0.66583100

O 2.31174200 1.45455000 -0.86096900

O 1.66941800 -1.24828700 -1.61443900

H 1.88177600 -1.79859500 -0.84910400

H 0.73583000 -1.43117400 -1.78022500

O 0.03762300 2.43009700 0.52332400

H -0.85339200 2.46344000 0.15382300

H -0.04841200 1.79536200 1.25082700

S -2.16135100 -0.40343900 -0.30536200

O -2.44066000 1.01010700 -0.19421900

O -1.15970700 -0.77063200 -1.28719700

TS1-2W:

N -1.46599300 -1.21864300 -0.07431900

O -1.95740200 -2.28830200 -0.18810700

O 0.38048400 -1.20564800 -0.90772800

O -1.99139700 -0.23432100 0.41914300

N -0.55040100 1.35740900 0.38954400

O -1.01123600 2.27076600 0.72199100

O 0.20847600 0.43584600 2.46311900

H -0.35213300 -0.20965900 2.90250200

H 1.02432600 -0.03746100 2.23373700

O -0.84120200 1.52024200 -1.97705500

H 0.11488400 1.41352500 -2.07661400

H -1.23002200 0.75967900 -2.41850900

S 1.64512800 -0.49208300 -0.48294000

O 1.62887500 0.95423300 -0.78590100

O 2.11285800 -0.83083200 0.86445600

IM1-2W:

N 0.77420300 -0.44318300 1.18138100

O 1.57568700 -0.82507800 1.97160500

O 2.76074800 -0.12304100 -0.52510800

O -0.44056500 -0.40560100 1.38730800

N -2.06636800 0.05890800 -0.07170800

O -2.90782200 -0.06699600 0.57414600

O -1.63349500 2.31849600 -0.09421700

H -1.68697200 3.01725500 0.56297100

H -0.68626700 2.22645500 -0.31091900

O -1.80361900 -2.02189200 -1.08609000

H -0.91563600 -1.75217700 -1.37462500

H -1.72642100 -2.92801000 -0.77752300

S 1.36753400 0.19861300 -0.56220900

O 0.49254600 -0.60285100 -1.40555100

O 0.97901100 1.59553700 -0.43112700

IM1'-2W:

N 1.38420100 -0.55730100 -0.90017200

O 2.41095800 -0.44066700 -1.49140100

O 1.91489500 1.74563400 0.29220800

O 0.54144800 -1.42401400 -1.07327600

N -2.12874400 -0.51328000 0.13777700

O -2.15307300 -0.88373600 -0.86313300

O -0.96515800 -2.02188700 1.33678600

H -0.35394300 -2.43092100 0.70948500

H -0.37766800 -1.44503700 1.85397500

O -2.91854100 1.70487700 -0.53222200

H -2.03374600 2.05230100 -0.35304300

H -3.54121700 2.40612000 -0.33033300

S 0.97257000 0.68824300 0.49285700

O -0.42788300 0.97242200 0.11448300

O 1.09201100 -0.16516600 1.66292800

TS1'-2W:

N 1.59098600 -0.81245200 -0.58091600

O 2.56517000 -0.83448700 -1.23349000

O 2.03812600 1.75135900 -0.01983000

O 0.87302700 -1.86324000 -0.46718200

N -2.34083500 -0.73894000 0.43762400

O -2.59179200 -1.11481500 -0.57333000

O -1.01608900 -1.58057100 1.01375900

H -0.02110400 -1.70345600 0.22442900

H -0.72066800 -1.07199700 1.78659700

O -2.97428500 1.67909100 -0.70133800

H -2.03032800 1.87292000 -0.71920700

H -3.41930000 2.47801800 -0.98852500

S 0.96545800 0.88872500 0.35709800

O -0.29962300 0.98487300 -0.34637700

O 0.90459400 0.36087100 1.70106100

SO_3_-2HONO-H_2_O-1:

N 0.99677200 -1.46698800 -0.17894000

O 1.77848000 -2.27793300 -0.47902900

O 2.93887600 0.34302600 0.28935900

O -0.24135700 -1.86713000 -0.02233900

N -3.25988100 -0.12180000 -0.03006900

O -3.51621100 -1.25151400 0.07463700

O -1.92860800 0.11959300 0.29293700

H -0.81737400 -1.07383300 0.19764300

H -1.75179200 1.08847700 0.10614100

O -1.34399500 2.54023200 -0.45450500

H -0.50931000 2.38928600 -0.92042900

H -1.19735000 3.26908300 0.15387900

S 1.54135400 0.59707300 0.15710100

O 1.03423400 1.11771600 -1.09260100

O 0.71057200 0.76292500 1.31806700

RC2-2W:

N 1.18827600 -0.59850900 -1.03852700

O 1.17700400 -1.77149800 -1.30560900

O 0.16984900 0.11583600 -1.04636400

O 2.29156500 -0.02657100 -0.70373000

N 2.07487800 1.13528600 0.82194900

O 1.05598300 1.18296000 1.17116500

O -0.25161400 2.87083600 -0.26975100

H -1.16171400 2.79950700 0.03476500

H -0.19177700 2.18524700 -0.94523200

O 1.09865800 -1.39953400 1.65342300

H 1.52763500 -2.15441800 1.24011000

H 0.15514300 -1.60238200 1.63948800

S -2.17495600 -0.56810100 -0.22335200

O -2.42239000 0.84047200 -0.02589300

O -1.66556300 -1.29947200 0.91682800

TS2-2W:

N -0.49947700 1.52911700 -0.76030400

O -0.14426000 2.66151300 -0.79966600

O 0.94319100 0.16617100 -1.39779000

O -1.63423400 1.16417600 -0.47098900

N -2.07192100 -0.58416300 0.84434000

O -1.27298800 -0.87291900 1.49412200

O -1.83753900 -1.80492200 -1.05026200

H -0.90703700 -2.06990400 -0.97762800

H -1.85419100 -1.09635100 -1.70366600

O 0.18860400 1.13660500 2.00795700

H 0.15462700 2.08952300 1.88189300

H 1.11814100 0.89725200 1.88180600

S 1.57787400 -0.71795000 -0.37982000

O 0.65609300 -1.76197200 0.12108800

O 2.38141800 -0.05715100 0.64634800

IM2-2W:

N -0.63673200 0.26453100 1.16226900

O -1.47772900 0.86242200 1.75942200

O -2.02396800 -1.62352500 -0.04700100

O 0.52575700 0.13084600 1.52826100

N 2.35881400 0.48595800 0.14796400

O 2.13665300 0.68808600 -0.87394400

O 2.45972000 -1.71377900 0.25398000

H 1.56830900 -1.82615100 -0.12942700

H 2.37977200 -1.98618400 1.17394300

O 0.28600100 2.40798600 -0.57813800

H -0.01445600 3.17961100 -0.09214600

H -0.50317300 2.05432000 -1.01821900

S -1.15241100 -0.57996100 -0.50600400

O 0.18126400 -0.98184100 -0.96325300

O -1.71850600 0.55534800 -1.20554100

IM2'-2W:

N 1.36786900 1.07385400 0.00003100

O 2.49460500 1.45619600 0.00104600

O 2.32746900 -1.39190600 -0.00142000

O 0.36505500 1.77417100 -0.00061700

N -2.69618100 -0.24388600 0.00105700

O -2.26922400 -1.22774100 0.00060800

O -1.80696500 0.81347400 -1.70679400

H -0.99112400 0.28456200 -1.80304800

H -1.48515800 1.68280400 -1.44011200

O -1.80464600 0.81296400 1.70737200

H -1.48183900 1.68151300 1.43931200

H -0.98940300 0.28299600 1.80316800

S 1.02063900 -0.81334600 -0.00048300

O 0.21586200 -0.91360000 -1.22088800

O 0.21728000 -0.91457400 1.22079000

TS2'-2W:

N 1.04639300 1.22213300 -0.01967000

O 1.88622300 1.90868900 0.44280500

O 2.75087800 -0.70371500 -0.67067900

O -0.03895900 1.71812600 -0.42796500

N -3.08485200 0.31574200 -0.13080400

O -3.56485600 -0.69139000 -0.06573300

O -1.83235800 0.28569600 -1.14455900

H -1.43697900 -0.61206500 -1.17720900

H -0.93925600 0.95464300 -0.77493000

O -1.33293100 -0.12453400 1.71561500

H -1.39211400 0.43659900 2.49254800

H -0.52094400 -0.64058000 1.83034300

S 1.42910800 -0.76407500 -0.13193000

O 0.34067100 -1.14010200 -1.01718900

O 1.25293000 -1.08758400 1.26688500

SO_3_-2HONO-H_2_O-2:

N 0.88218500 1.33896800 0.19639700

O 1.46007100 2.13199900 0.82439400

O 2.86979600 -0.23657400 -0.87138500

O -0.24727400 1.74886500 -0.35432500

N -3.34721600 0.23875500 -0.63667900

O -3.79486800 -0.24745400 0.33094700

O -2.08638300 -0.21205800 -0.93743900

H -1.77862900 -0.79929900 -0.20285000

H -0.68702800 0.96856600 -0.77028100

O -1.04293400 -0.77078400 1.49345200

H -1.66947200 -0.78661700 2.22179500

H -0.23060200 -1.19523000 1.79686600

S 1.66462000 -0.69298100 -0.25763000

O 0.51879600 -1.01394700 -1.06563900

O 1.69617300 -1.16802100 1.09980900

t-ONONO_2_-(H_2_O)_3_:

N -0.44621800 -0.78534000 0.84124100

O -1.49298500 -0.67295100 1.43498100

O -0.31664500 -1.47081400 -0.18328600

O 0.58867100 -0.14040300 1.26077600

N 1.19267700 0.88084100 -0.26585400

O 1.92129300 1.59344300 0.10007800

O 2.45359200 -1.13800500 -0.86277100

H 1.60921400 -1.56365700 -0.65030700

H 2.76526600 -1.55119100 -1.67052900

O -2.37069100 -0.05330000 -1.52976800

H -1.86043300 -0.82143100 -1.23386100

H -3.28505700 -0.25088100 -1.31461800

O -0.97868700 1.92431400 -0.12218600

H -1.54736200 1.38381400 -0.70119300

H -1.34322500 1.79656600 0.76021200

c-ONONO_2_-(H_2_O)_3_:

N -0.00237000 -1.21036700 -0.19121300

O -1.06293500 -1.31039800 -0.80402100

O 0.99360000 -1.85675300 -0.45919800

O 0.05426900 -0.36229800 0.78063100

N -0.80319500 1.34711600 0.29367100

O -1.16222100 1.33974900 -0.72253800

O 1.48870200 1.88232600 -0.43167600

H 2.05412700 2.58316200 -0.76188000

H 2.08934300 1.20685300 -0.05533800

O -3.06053100 0.10754100 0.64734600

H -2.64021300 -0.67093000 0.25492100

H -3.71287800 -0.21725800 1.26999400

O 3.04902400 -0.14680600 0.58910500

H 2.91704700 -0.19682400 1.53937200

H 2.53226800 -0.88912700 0.23853600

RC1-3W:

N 1.08070200 0.25702500 -1.25854400

O 2.12196300 0.55118700 -1.80302700

O 0.85909300 -0.92209900 -0.88556300

O 0.19912900 1.13619700 -1.03792600

N -0.56358300 1.45393300 0.95445400

O -0.26795400 2.46222400 1.16944500

O -2.43846000 1.87170900 -0.47397800

H -1.90383500 1.92386400 -1.27557700

H -2.75939200 0.96000200 -0.49218100

O 3.26373300 -0.72246400 0.60294200

H 2.73529900 -1.16851900 -0.07406500

H 3.84717300 -0.14352200 0.10446900

S -1.28935100 -1.85194800 -0.08553500

O -0.98794200 -1.59960700 1.30902700

O -2.24710500 -0.94618000 -0.68611800

O 1.07076400 0.25141800 2.00057900

H 1.92512300 0.00008300 1.59595200

H 0.57963700 -0.57653100 2.07554900

TS1-3W:

N 1.74224800 -1.11645000 -0.42939400

O 2.79490900 -1.34215500 -0.92165900

O 0.83425700 0.53504900 -1.32078400

O 1.27221100 -1.67843800 0.54809300

N -0.70793400 -0.84748100 0.84534100

O -1.27608700 -1.48475600 1.49842500

O 0.55259000 0.45398300 2.45869400

H 1.41659300 0.03356800 2.51347800

H 0.73107400 1.34440600 2.12382500

O -1.12547000 -1.66147200 -1.29439000

H -2.01904100 -1.29534300 -1.37990100

H -0.55150300 -1.10208900 -1.83077900

S 0.20184800 1.69460500 -0.60371000

O -1.17863900 1.44579700 -0.14131700

O 1.06436800 2.35324000 0.37777400

O -3.30855900 -0.23568400 -0.43088700

H -2.83673200 0.60946200 -0.40180900

H -4.24679400 -0.04068100 -0.46866400

IM1-3W:

N -0.60658000 -0.02157700 1.17531500

O -1.46425600 0.07775300 1.99743300

O -2.46173500 -0.43415200 -0.62130000

O 0.56244000 0.30151500 1.35255100

N 2.18190900 0.34702900 -0.19570200

O 2.98946800 0.65777400 0.43487700

O 2.14633600 -2.05604700 -0.04445500

H 2.40183500 -2.68945200 0.63082800

H 1.22327200 -2.27360300 -0.25720700

O -1.47624700 2.35029200 -0.05834200

H -2.09185400 1.73083400 -0.47405900

H -1.91774100 2.65225800 0.74006900

S -1.06945800 -0.80236000 -0.55057600

O -0.15357300 -0.08785300 -1.42064200

O -0.71785700 -2.18441900 -0.27675900

O 1.22366700 2.24027400 -0.92772800

H 0.39494200 2.37065800 -0.42920400

H 0.88761500 1.84780300 -1.74360000

IM1'-3W:

N -0.70058600 0.30507000 1.14158000

O -1.36294400 1.12448300 1.71485200

O -2.42312000 -1.55154100 0.36049900

O 0.46139900 0.04092300 1.37510300

N 3.03629300 0.07357700 -0.13707100

O 2.91271900 0.48716100 0.84708800

O 2.11475200 -1.98160800 0.05159200

H 1.15346000 -1.84144600 -0.04131000

H 2.33359900 -2.67689700 -0.57547400

O -0.28526400 2.57794700 -0.69486100

H -1.10482000 2.13016000 -0.96182600

H -0.47561100 2.96590000 0.16442700

S -1.54196400 -0.64377200 -0.31523700

O -2.10285100 0.47317100 -1.04972500

O -0.33302800 -1.22472000 -0.92004600

O 1.57450000 0.75055500 -1.42793800

H 1.01060900 1.50240100 -1.12718200

H 0.95491500 -0.00126500 -1.49890700

TS1'-3W:

N -0.57238200 0.06685000 1.17252500

O -1.23351900 0.84298500 1.76795000

O -2.41931800 -1.60365200 0.30616200

O 0.57832000 -0.24108600 1.58764600

N 3.16071800 -0.19837000 -0.53828800

O 3.59619000 0.40384900 0.29150800

O 2.12796400 -1.29772700 0.08877300

H 1.30585000 -0.78944600 0.80085400

H 1.59591400 -1.62536300 -0.65961700

O -1.00781700 2.95529800 -0.38614500

H -1.66344900 2.26996600 -0.57400400

H -1.27122300 3.35181700 0.44752000

S -1.44901100 -0.84107000 -0.41992000

O -1.87122600 0.35305000 -1.11411400

O -0.29932500 -1.53575600 -0.96466900

O 1.02767000 1.16605600 -0.98123800

H 0.52812400 1.96180600 -0.72485000

H 0.57910300 0.86484600 -1.77781600

SO_3_-2HONO-2H_2_O-1:

N -0.87201200 -1.31187900 0.51899800

O -1.56468900 -1.98844000 1.17321900

O -2.46294900 -0.58280300 -1.55582800

O 0.39498200 -1.61958400 0.49145800

N 3.46214600 -0.23773800 -0.30603300

O 3.66116800 -1.38503700 -0.35399500

O 2.12274700 0.04786500 -0.50116600

H 0.92257100 -0.91882500 -0.00731700

H 1.99129300 1.04068700 -0.35230600

O -0.10154500 1.18141100 1.93748400

H -1.05391500 1.32116900 1.84496300

H 0.08347300 1.12600000 2.87865700

S -1.72866000 0.26815600 -0.67454200

O -2.41389100 0.91382500 0.41640000

O -0.46800400 0.82044300 -1.09905000

O 1.54176500 2.48051000 0.08645000

H 0.92598500 2.21714200 0.79411300

H 0.98153100 2.82512500 -0.61596900

RC2-3W:

N -0.15832000 -0.21380500 1.57750300

O -1.20455800 -0.08469800 2.17783200

O 0.09372500 -1.30607500 0.96284500

O 0.68872100 0.69892000 1.51852100

N 1.25734200 1.91213700 -0.36126300

O 0.40375600 2.12744500 -0.97093000

O 2.32878100 0.37328200 -1.30833100

H 1.64439700 -0.16186500 -1.73848700

H 2.69163000 -0.21291300 -0.60048300

O -1.75105100 2.01174300 0.30526500

H -1.69569600 1.81062400 1.24741200

H -2.32176400 1.31432100 -0.03963200

S -1.20040700 -1.35130200 -1.06021100

O -0.27180600 -0.47631700 -1.76823000

O -2.39801600 -0.69570100 -0.58584400

O 2.85027800 -1.42654000 0.58543200

H 3.36019700 -1.31012400 1.38985700

H 1.91595800 -1.48400800 0.85854100

TS2-3W:

N 0.03899700 1.75378400 -0.34704700

O 0.86483600 2.61225800 -0.34504600

O 0.46939700 0.08987200 -1.52317200

O -0.90764800 1.68906600 0.42255700

N -1.46347400 -0.11893700 1.80431100

O -0.59514700 -0.63637900 2.15883500

O -2.36716700 -1.33506300 0.33390400

H -1.61281600 -1.86733100 0.03187000

H -2.56088500 -0.73737800 -0.42446500

O 1.57272600 0.62600900 1.80641500

H 1.76548500 1.55544600 1.64356100

H 2.27683800 0.14279800 1.35254000

S 1.16201500 -1.09014900 -0.89938900

O 0.30062500 -1.84470400 0.03476200

O 2.52542000 -0.85742200 -0.43394800

O -2.41435300 0.32487100 -1.81403200

H -2.62014100 1.23121900 -1.56682900

H -1.45890200 0.32564400 -1.96950500

IM2-3W:

N -0.78870800 -0.03344200 1.13321600

O -1.75472500 0.17508400 1.79850700

O -0.52529700 -2.10150200 -0.41009200

O 0.37077600 0.12838600 1.48646100

N 1.95272200 1.52482600 0.29537400

O 1.43570400 2.21711100 -0.35000600

O 2.50503100 -0.00904900 -0.79602100

H 1.67943700 -0.12392100 -1.31324400

H 2.50981600 -0.80805300 -0.19571200

O -1.12186300 2.46219400 -0.10142800

H -1.91824300 2.59676100 0.41996500

H -1.41800000 2.04662000 -0.91902500

S -1.01340200 -0.75131400 -0.63434300

O -0.09142900 0.11253100 -1.38491000

O -2.41187700 -0.53320200 -0.86952700

O 2.10235500 -2.17125000 0.66382800

H 1.90087600 -1.95012700 1.57847700

H 1.24305200 -2.42235800 0.28438500

IM2'-3W:

N -0.78833700 -0.03676500 1.13323400

O -1.75551100 0.16592100 1.79867800

O -0.52470000 -2.10371600 -0.41138700

O 0.37025900 0.12946300 1.48753100

N 1.94648800 1.53159300 0.29665900

O 1.42708400 2.22037600 -0.35035100

O 2.51091800 -0.00125100 -0.79338700

H 1.68754500 -0.11939300 -1.31318300

H 2.51586000 -0.80019500 -0.19349100

O -1.13354700 2.45495700 -0.10029900

H -1.92518300 2.59268600 0.42747400

H -1.43809200 2.03958900 -0.91491200

S -1.00966200 -0.75250900 -0.63581300

O -0.08490400 0.11052000 -1.38348500

O -2.40730300 -0.53153300 -0.87410300

O 2.10528300 -2.16597900 0.66428900

H 1.89747000 -1.94517400 1.57761100

H 1.24929300 -2.42123600 0.28035400

TS2'-3W:

N 0.81594500 -0.42793200 1.10865400

O 1.51124200 -1.22665300 1.64881900

O 1.32779400 1.73494900 -0.21074300

O -0.24535400 0.01311100 1.51140700

N -2.76361400 -0.22669400 0.19199900

O -2.73142100 -1.22794600 -0.31713100

O -2.08261500 0.87834800 -0.70151800

H -1.33693200 0.44259800 -1.19095300

H -1.56313500 1.79331700 0.00903000

O -0.37209300 -2.56160700 -0.27124000

H 0.15400300 -3.31915100 -0.00470300

H -0.00334400 -2.26036500 -1.10810200

S 1.38330100 0.30698100 -0.55947600

O 0.28791700 -0.11126900 -1.44388200

O 2.66819500 -0.28038600 -0.77553000

O -0.96032000 2.57204100 0.60114400

H -0.96204500 2.28479600 1.52580500

H -0.01442800 2.40477600 0.28535500

SO_3_-HONO-(H_3_O^+^)(NO_2_^-^)-2:

N 2.10314400 -0.63550700 -0.84558800

O 3.08105400 -0.41215100 -1.47306100

O 1.44636900 -0.36281400 1.66433400

O 1.36256100 -1.61105400 -1.03123200

N -3.78765800 -0.78988700 0.13466500

O -4.54866600 0.03802300 -0.17356100

O -2.47256700 -0.36365500 0.06366500

H -2.39147500 0.60158700 -0.28129900

H -1.30065500 -1.26735600 0.43609300

O -2.04307500 1.98945000 -0.82762000

H -2.50518600 2.79287400 -0.57701100

H -1.10085900 2.09799100 -0.62476700

S 1.56309200 0.58850800 0.56126200

O 0.27225900 0.98167900 0.00511200

O 2.63304700 1.53364600 0.57416800

O -0.49565200 -1.84811200 0.66662100

H 0.19113000 -1.80794700 -0.10377200

H 0.00653400 -1.41562400 1.39959100

SO_3_-2HONO-2H_2_O-2:

N 1.33424100 -0.50574000 1.12773000

O 1.78837700 -1.50709100 1.53307600

O 1.95790300 1.58590700 -0.42761200

O 0.51852600 0.15428700 1.86565100

N -3.90163400 0.48822300 -0.19119900

O -4.35495500 -0.58546100 -0.30952900

O -2.53606400 0.49014800 -0.10097900

H -2.17736900 -0.44547800 -0.15322500

H -1.31915200 1.76476000 0.32356600

O -1.22466900 -1.80314800 -0.24984000

H -1.57400900 -2.69263300 -0.34599800

H -0.56919700 -1.65661100 -0.94591800

S 1.83239900 0.18858300 -0.76768500

O 0.60053700 -0.20266700 -1.42204700

O 3.00169500 -0.60848800 -0.95309600

O -0.49109300 2.06328500 0.74451200

H 0.10794700 1.02767500 1.34146200

H 0.10309900 2.41341200 0.06624800

t-ONONO_2_-NH_3_:

N -1.03745300 -0.46252000 -0.03506800

O -1.84630800 0.08427200 -0.73418800

O -1.13970100 -0.74500700 1.12848100

O 0.12843000 -0.78955700 -0.65285800

N 1.36123600 -0.40757500 0.36855700

O 2.30854500 -0.50224500 -0.21246200

H -0.20933500 2.15761600 -0.74104700

H -0.43889700 2.15802600 0.87386000

N 0.27008400 2.07411500 0.15094700

H 0.88342200 2.87651900 0.24434300

c-ONONO_2_-NH_3_:

N -1.07399900 -0.16213700 0.05967600

O -1.70131800 0.86823200 0.04690400

O -1.10163200 -1.03201300 -0.78620800

O -0.24984000 -0.35204500 1.08723900

N 1.39812400 -0.70557800 0.51197500

O 1.43679500 -1.16203500 -0.48515000

H 0.60934600 2.08855600 0.56463000

H 0.45773400 2.04890300 -1.04975000

N 1.10040500 1.84112100 -0.29030700

H 1.88916100 2.47159100 -0.38656700

RC1-1A:

N -1.63099800 -0.80521400 0.10401200

O -2.58556800 -1.20085900 0.70823400

O -0.89387500 -1.49144300 -0.59711800

O -1.33601200 0.48030200 0.20937900

N -0.57837000 1.11458000 -1.31310000

O -0.40090100 2.17812600 -1.15119700

H -0.41758400 2.32045200 1.85970000

H 0.86202100 1.34222800 1.64689300

S 1.67116800 -1.12489600 -0.12318800

O 1.70647500 -0.02852100 -1.06593900

O 1.46590400 -0.77132400 1.26149700

N 0.50554600 2.27132300 1.44091600

H 1.09545900 2.94057400 1.92277900

TS1-1A:

N -1.44161300 -1.13142800 -0.13475200

O -1.96697700 -2.02031700 0.44375600

O 0.54273800 -1.54025300 -0.64566900

O -1.94812300 -0.04466900 -0.37953500

N -0.50516100 1.35469100 -1.25342700

O -0.70481200 2.28200000 -0.74498700

H -1.29521900 1.17740700 2.11112600

H 0.33180300 1.06678300 2.01669900

S 1.56572500 -0.58413500 -0.14241800

O 1.55678500 0.70016100 -0.89324900

O 1.66211300 -0.46228700 1.30859200

N -0.46023000 1.67672500 1.81867700

H -0.37294500 2.48496000 2.42611100

IM1-1A:

N -0.65444900 1.23746400 -0.12427300

O -1.43691600 2.13345300 -0.07411400

O -2.66302300 -0.44815500 0.26106200

O 0.55201300 1.34316600 -0.29310400

N 2.83672000 0.17443600 -0.36929200

O 3.06575800 0.57133900 0.65273600

H 2.45526500 -2.05339100 -0.33567800

H 1.29693800 -1.21393700 0.61834700

S -1.25039100 -0.57049900 0.07779800

O -0.74377700 -1.11399100 -1.18336000

O -0.42394900 -0.94623900 1.23029500

N 1.85981000 -1.23002800 -0.25733900

H 1.15863200 -1.19437300 -1.01922700

RC2-1A:

N -0.87295500 1.26010600 -0.13086400

O -0.67501100 1.60450000 1.02790800

O 0.07630200 1.08652000 -0.92902400

O -2.03538200 0.99337000 -0.53350400

N -1.77464400 -1.19456600 -0.82642500

O -0.77081500 -1.33829700 -1.22583100

H -2.22220700 -0.29213800 1.45426300

H -0.74528900 -1.05954800 1.54377700

S 1.99273500 0.10848800 0.36190300

O 2.76549500 -0.26620500 -0.79358500

O 1.11627100 -0.93216700 0.88708100

N -1.71928500 -1.15355400 1.25046200

H -2.16295400 -1.94979400 1.69494600

TS2-1A:

N 1.28446400 -1.05285600 -0.41906100

O 1.66492400 -1.99835200 0.18206200

O -0.60582200 -1.11561000 -1.00868600

O 1.96561000 -0.12219200 -0.80930300

N 1.25236400 1.92635300 -0.74781000

O 0.55304200 2.07001300 0.05184400

H 1.42872500 -0.33555700 2.20112800

H -0.16412100 0.02095900 2.13730800

S -1.66692100 -0.28658500 -0.32261000

O -1.48585300 1.16071700 -0.56540000

O -1.91772300 -0.65068600 1.07239400

N 0.76922200 0.43134600 2.09791600

H 0.87035100 1.01493700 2.92271900

IM2-1A:

N 0.69020300 1.14822300 -0.45194400

O 1.53699600 1.98252700 -0.39104800

O 2.39763200 -0.60146000 0.58548800

O -0.42532600 1.27892700 -0.93381700

N -1.51354900 -0.91177000 -1.22647800

O -2.09952100 -1.10662900 -0.35176600

H -2.37914900 1.83193600 0.53621600

H -1.81073000 0.73529200 1.60808600

S 0.99593600 -0.57624500 0.31515500

O 0.51679000 -1.38665400 -0.83036200

O 0.03144500 -0.53164400 1.39905200

N -2.60601900 0.96751600 1.01768100

H -3.40365500 1.14436600 1.61803400

t-ONONO_2_-H_2_O-NH_3_:

N 0.69403900 -1.08273400 0.14700600

O 1.88660900 -1.22265600 0.33101300

O 0.04334800 -1.68273000 -0.68676200

O 0.07408400 -0.19861400 0.89575300

N -0.72311200 1.03115400 -0.26040600

O -0.94781800 1.91621300 0.33531700

O 1.80514800 1.60400000 -0.44935800

H 2.17227300 0.90700200 0.11044600

H 2.43826800 1.71647600 -1.16170700

N -2.68489300 -0.46074500 -0.16529600

H -2.87429600 -0.70064000 0.80253800

H -2.06755700 -1.18001900 -0.53501600

H -3.56188700 -0.48625400 -0.67307600

c-ONONO_2_-H_2_O-NH_3_:

N 0.51853900 -1.08154400 0.01058700

O 1.65192400 -1.49080300 -0.12631100

O -0.18928500 -1.29777600 0.98629500

O 0.02220800 -0.32817800 -0.93200800

N -0.70278400 1.28159400 -0.14333500

O -0.51336900 1.32659800 0.92540700

H -3.72827400 0.07676100 -0.19546700

H -2.38528300 -0.79995900 0.21314100

O 2.03817400 1.48151000 -0.19984600

H 2.17134200 0.65857600 -0.68658500

H 2.88370900 1.67455900 0.21009000

N -2.73412400 -0.03425400 -0.35903900

H -2.59012800 -0.30130900 -1.32696300

RC1-W-A:

N -0.55367000 -0.91778600 -1.21373900

O -0.49012000 -2.10006100 -0.93401800

O 0.49842700 -0.25386800 -1.45480300

O -1.64400900 -0.30451700 -1.23369300

N -1.34411000 0.99592600 0.62837100

O -2.32265400 1.37748900 0.89557300

O -1.59036700 -1.27607800 1.65124000

H -1.79857300 -1.85276800 0.90584300

H -0.63964200 -1.39447800 1.77098100

S 2.12264100 -0.28862100 0.29279600

O 2.33973900 1.13811900 0.14999000

O 1.19671400 -0.66009700 1.34818100

N -0.27806700 2.35738100 -0.59805900

H -0.65414800 3.26189400 -0.86333000

H -0.19233500 1.73588900 -1.40275400

H 0.65154500 2.45085600 -0.19124200

TS1-W-A:

N -1.36209200 -1.27370800 -0.36356000

O -1.65227800 -2.40293600 -0.58040800

O 0.45941600 -0.80784000 -1.19053200

O -2.05348800 -0.44735400 0.20232600

N -0.68048400 1.26818800 0.59362200

O -1.11499800 1.99173900 1.27042500

O 0.10759000 -0.31104300 2.36674000

H -0.43062500 -1.08174400 2.56641800

H 0.97333300 -0.66165300 2.10845300

S 1.68314700 -0.20401500 -0.53482700

O 1.57778300 1.25325200 -0.32537000

O 2.19747700 -0.96579600 0.60644400

N -0.95015300 1.85334800 -1.55119000

H -1.74545200 2.31942700 -1.97418700

H -0.85754900 0.91625800 -1.93475200

H -0.09297200 2.35698100 -1.75778400

IM1- W-A:

N -1.22298800 -1.00880200 -0.57575800

O -2.25666800 -1.42514600 -0.99270800

O -2.54854900 1.07279700 0.36869300

O -0.14428900 -1.59278500 -0.64888900

N 1.85819200 -0.35493100 -0.22319600

O 2.54014600 -0.78034300 -0.94965200

O 2.38485900 2.03516400 -0.44166300

H 2.75360900 2.91987100 -0.44391500

H 1.41984300 2.12971000 -0.48756200

S -1.16332000 0.71510800 0.30565300

O -0.49649300 0.31034400 1.53698100

O -0.31149200 1.42452900 -0.64389800

N 1.69952200 -1.48813100 1.50243500

H 2.46520400 -1.52421900 2.16714300

H 1.37880100 -2.42057400 1.25882700

H 0.91245800 -0.93994400 1.85976900

IM1'- W-A:

N 0.75342000 0.20500100 1.22578000

O 1.47242800 0.70495000 2.03265700

O 2.76259500 0.29411900 -0.49034200

O -0.40845500 -0.13417100 1.42506100

N -2.14694500 -0.14518800 -0.23914600

O -2.95931100 0.05166700 0.45314300

O -1.71862000 2.27672700 -0.73710200

H -1.65763700 3.22707000 -0.62783700

H -0.84691300 1.97444100 -1.03491300

S 1.39775400 -0.13221500 -0.56709900

O 1.12214200 -1.56331300 -0.63655200

O 0.45196200 0.69317700 -1.30880100

N -1.63105100 -2.12006700 -0.21256600

H -2.14063800 -2.72593800 -0.84851900

H -1.69065900 -2.46908000 0.74044400

H -0.63811700 -2.05452400 -0.47859600

TS1'- W-A:

N 0.87170700 1.26054800 0.10153500

O 1.57563400 2.08492500 0.56204900

O 2.94608500 -0.27191100 -0.56781600

O -0.34490400 1.52608700 -0.14189200

N -3.06697400 -0.49986300 -0.37429300

O -3.65041200 0.42442600 -0.14451500

O -1.74994900 -0.08417600 -1.24250900

H -1.20036800 -0.88576300 -1.33283700

H -1.01758500 0.67552300 -0.68661800

S 1.62129700 -0.62316600 -0.16447600

O 1.38885100 -1.09988400 1.17747100

O 0.68643100 -1.06922100 -1.18065800

N -1.56571000 -0.70975200 1.78190500

H -2.13329900 -1.28550100 2.39632500

H -1.42860700 0.17627400 2.26136000

H -0.64793400 -1.14837900 1.73231800

SO_3_-HONO-NH_4_NO_2_-1:

N 1.11493300 1.08892100 0.68689900

O 1.70359600 2.11860100 0.62845900

O 2.63656700 0.20257400 -1.28783100

O 0.19766000 0.83655500 1.46605400

N -3.33749600 0.68067800 -0.73715900

O -4.27038300 0.42567100 -0.08914400

O -2.28306600 -0.16526700 -0.43619600

H -1.52861900 0.09965100 -0.99768500

H -0.50389500 -0.76067200 1.42572300

S 1.56595200 -0.34697900 -0.51805400

O 1.83001700 -1.39783000 0.45318200

O 0.25340400 -0.50076600 -1.16572000

N -0.78048500 -1.74558000 1.19789900

H -0.68605400 -1.80694000 0.17576100

H -1.74548800 -1.92297200 1.46937700

H -0.11220400 -2.38184700 1.63180100

RC2- W-A:

N 1.11532700 -0.56377100 -1.11417200

O 1.33669600 -1.75448300 -1.04889500

O -0.03331200 -0.14892000 -1.51199000

O 1.94745000 0.29456700 -0.78271100

N 0.97396400 1.76464100 0.95857400

O 0.32979800 1.12585900 1.55999200

H -1.00526000 2.71415600 -0.35254600

H -0.01241700 1.68158900 -1.21478800

O 1.50584000 -1.17204800 1.82049500

H 2.09386700 -1.45725400 1.11236700

H 0.68353000 -1.64387900 1.64493400

S -1.91177600 -0.70544700 -0.18438000

O -2.11569700 0.70575200 0.10636400

O -1.21821100 -1.44882200 0.84703400

N -0.03968000 2.49707600 -0.58639900

H 0.46089700 3.29166800 -0.96822700

TS2- W-A:

N -0.06246500 1.72007200 -0.72087100

O 0.43389700 2.66719500 -0.18882300

O 1.19706100 0.18180400 -1.33522900

O -1.26461200 1.48406900 -0.72378400

N -2.36215700 -0.39043200 0.66812200

O -1.68271800 -0.85092700 1.38131000

H -1.27837400 -1.76183000 -1.09932800

H -1.81332600 -0.25567800 -1.60130300

O 0.13419500 0.78240300 2.05455800

H 0.26902600 1.71444900 1.85120600

H 1.02089800 0.39623400 2.03898400

S 1.51623100 -0.92633600 -0.38283500

O 0.37493700 -1.82869800 -0.11804900

O 2.30643600 -0.57446200 0.79541200

N -2.08201600 -1.13167300 -1.14966400

H -2.90501800 -1.54865800 -1.57046200

IM2- W-A:

N -0.79461100 -0.10168100 1.19969900

O -1.39684100 0.76370000 1.77178200

O -2.05759500 -1.46783300 -0.68209500

O 0.02937100 -0.84327200 1.69221500

N 2.00923400 0.41382900 -0.02289900

O 2.59618600 0.80072200 -0.84511700

H 1.58994600 -1.94245100 -0.23432400

H 2.15049400 -1.76021200 1.30245100

O 0.50292100 2.42361300 0.07609100

H 0.14680300 2.72280000 0.91795600

H -0.28457100 2.23215000 -0.45884200

S -1.14394100 -0.35883300 -0.67957300

O 0.23398900 -0.65447800 -1.11056300

O -1.62387100 0.95488400 -1.05617000

N 2.38385900 -1.62810600 0.32323300

H 3.23772900 -2.11793100 0.07655600

IM2'- W-A:

N -0.91231700 -0.12872800 1.19501200

O 0.25539300 -0.28482400 1.52588600

O -2.60841900 -0.03775600 -0.83589800

O -1.84085400 -0.03527100 1.93721300

N 2.24712400 -0.07465500 -0.02235800

O 2.02210600 0.18062600 -1.04759300

H 1.00572200 -2.19654900 -0.24152600

H 1.73458700 -2.18184400 1.25163100

O 1.66157100 2.41264000 0.38708800

H 1.55125000 2.96977400 1.15956000

H 0.78077300 2.31625700 -0.00498300

S -1.18321000 -0.01117400 -0.70642500

O -0.42537600 -1.19472400 -1.10880600

O -0.48185700 1.24473200 -0.95515700

N 1.89777600 -2.11194700 0.25235700

H 2.57042600 -2.80491200 -0.05882400

TS2'- W-A:

N -1.17850200 1.10039000 -0.41317800

O -0.12156400 1.55005100 -0.97155800

O -2.59270900 -0.89169400 0.65943700

O -2.09580600 1.81531200 -0.24380200

N 2.83383000 -0.19203100 -0.31102900

O 2.86334400 -1.16712300 0.23562000

H 1.04583800 0.98769900 1.79237300

H 1.38639800 2.36061500 0.95590600

O 1.55472300 -0.14319900 -1.33587100

H 0.70092700 0.74312700 -1.11217900

H 1.07191300 -0.99294800 -1.29423900

S -1.22284800 -0.85325800 0.26035100

O -0.18578400 -0.70702100 1.25652200

O -0.82381100 -1.49045600 -0.97802400

N 1.79317100 1.52168900 1.35677100

H 2.43385200 1.81633400 2.08598600

SO_3_-HONO-NH_4_NO_2_-2:

N 0.77409500 -0.80324100 0.99140600

O -0.15364300 -0.29231300 1.61205000

O 2.39313100 -0.61292200 -1.09547800

O 1.29430200 -1.83939000 1.24734200

N -2.97442900 -0.75370300 -0.19309600

O -2.74768400 0.40532800 -0.04178600

H -0.88796200 1.81562400 -0.37319100

H -0.05720800 2.83003200 0.66071000

O -2.04956300 -1.40119300 -0.88464500

H -0.71105300 1.38245700 1.18138900

H -1.31195800 -0.77980400 -1.11812900

S 1.37173000 0.21010500 -0.53106500

O 1.71607700 1.46402300 0.12020000

O 0.07883800 0.28995900 -1.24317400

N -0.86823900 2.21565000 0.57777600

H -1.75114000 2.67114400 0.79757500

t-ONONO_2_-2H_2_O-NH_3_:

N -0.53051300 -1.20777600 -0.51061500

O -1.42489400 -1.94605000 -0.14175800

O 0.65892200 -1.45950600 -0.39658800

O -0.86548000 -0.06854200 -1.04887800

N -0.31195000 1.30426600 0.18184800

O -0.72365400 2.23582900 -0.18985300

O -2.46756700 0.42997900 1.23028300

H -2.56061800 -0.34765600 0.66202600

H -2.81436300 0.16661800 2.08522700

O 1.82503500 1.25088000 -0.77552900

H 2.37445900 0.79721400 -0.09930700

H 1.58464700 0.53330500 -1.37328200

H 3.13638700 -0.24625000 2.09556400

H 2.41828600 -1.18306600 0.95524000

N 3.10395900 -0.44578400 1.10186200

H 4.01183200 -0.81583400 0.84145800

c-ONONO_2_-2H_2_O-NH_3_:

N 0.84466700 -1.07060200 0.04484000

O 1.93575900 -1.54324100 -0.20962700

O 0.28259800 -1.17903200 1.13236400

O 0.24321900 -0.37350600 -0.86723900

N -0.10493600 1.42459700 -0.09911000

O -0.03425900 1.40271200 0.97758200

O -2.49640500 1.37980300 -0.15198300

H -2.65411900 0.40219300 -0.19958400

H -3.07506000 1.78268200 -0.80201700

O 2.47771300 1.32387500 -0.35203200

H 2.58330900 0.46474500 -0.78142700

H 3.33442300 1.52610800 0.02982800

N -2.50187400 -1.36957300 -0.20958400

H -3.27518500 -2.01851900 -0.11754500

H -1.84708000 -1.54405400 0.55033700

H -2.00029300 -1.58900900 -1.06513100

RC1-2W-A:

N 0.51626700 -0.68155800 1.31266300

O 0.17134900 -1.84491700 1.44729900

O -0.33064500 0.23182800 1.16865100

O 1.73611000 -0.37182500 1.27077400

N 1.88820900 0.31541000 -0.84215300

O 2.92888800 0.27816600 -1.11314400

O 1.31641300 -2.02179600 -1.22496000

H 1.37690400 -2.46124400 -0.36721600

H 0.36523800 -1.98344800 -1.39575700

O 1.64360900 2.23341700 0.08996400

H 0.70580000 2.54690100 -0.06988300

H 1.60080400 1.82626700 0.96643500

S -2.25263700 -0.67860500 -0.11971100

O -2.80016600 0.63924000 -0.35424500

O -1.41365200 -1.19881800 -1.18414800

H -1.51176000 2.56288700 -0.89774400

H -1.35063500 2.56057900 0.70459900

N -0.97213800 2.97683200 -0.14269600

H -1.18578100 3.96858300 -0.12130700

TS1-2W-A:

N -1.03947200 -1.04580600 1.31879400

O -1.48999300 -1.18131400 2.40758900

O 0.23823500 0.56937500 1.27208800

O -1.23457200 -1.78254000 0.36675200

N -0.16224300 -0.80699900 -1.32954000

O -0.33153200 -1.27303700 -2.28341600

O -2.26410900 0.47916100 -1.32282400

H -2.95214300 0.09794300 -0.76994700

H -2.14127700 1.38240000 -0.99503800

O 1.59329500 -2.01800300 -0.55880900

H 2.25350000 -1.40243000 -0.14421100

H 1.26644200 -2.57693700 0.15206100

S 0.30346600 1.68776000 0.26438700

O 0.96535800 1.30378900 -0.99882100

O -0.92747200 2.46792100 0.12026800

H 3.09510400 0.62914500 -0.17027600

H 2.77431400 0.20203400 1.37709900

N 3.23424000 -0.10070300 0.52343000

H 4.22725600 -0.18457600 0.70871800

IM1-2W-A:

N -0.33389600 0.19750700 1.20150500

O -0.74476600 1.22715400 1.65611700

O -2.61749800 -0.03277700 -0.06372600

O 0.75176100 -0.29516400 1.47944900

N 2.14221600 -0.01814000 -0.38893800

O 3.12012800 -0.08979900 0.04168800

O 1.56604100 -2.26363200 -0.74166300

H 1.02308300 -2.53059400 0.01380800

H 0.89581600 -2.04152500 -1.40245400

O 1.54630800 2.08369100 -0.06307700

H 0.67023200 2.29427800 -0.52103900

H 1.38544900 2.28238500 0.86758200

S -1.36094600 -0.72493200 -0.16488700

O -0.52296000 -0.39915900 -1.31882900

O -1.24053700 -2.08339600 0.31512200

H -0.96560800 1.79059400 -1.90421000

H -1.59789800 2.33355300 -0.51203000

N -0.86941600 2.51814700 -1.19995200

H -1.07608100 3.41227700 -1.63240800

IM1'-2W-A:

N -0.33457000 0.20032300 1.20120100

O -0.74092000 1.23292800 1.65314100

O -2.61949300 -0.02428300 -0.06264000

O 0.74879800 -0.29649600 1.48068700

N 2.14142000 -0.02817700 -0.38756100

O 3.11869600 -0.10533200 0.04359100

O 1.55461000 -2.26996700 -0.74385600

H 1.00917800 -2.53737200 0.00970300

H 0.88634100 -2.04217300 -1.40474100

O 1.55845700 2.07661500 -0.06007700

H 0.68512800 2.29424900 -0.51960100

H 1.39610100 2.27270600 0.87085700

S -1.36487900 -0.71972900 -0.16415300

O -0.52639700 -0.39632600 -1.31861000

O -1.24772800 -2.07853900 0.31573900

H -0.95027900 1.79139300 -1.90361700

H -1.58330600 2.34617600 -0.51640500

N -0.85246100 2.52306900 -1.20381300

H -1.05400100 3.41539300 -1.64234300

TS1'-2W-A:

N -0.47896000 -1.15521000 0.84698500

O -0.93306300 -1.30948400 1.92249400

O -2.32090900 0.67639700 0.37630000

O 0.59257900 -1.72517300 0.50386000

N 3.00085400 0.33425400 -0.54108700

O 3.61896500 -0.27790200 0.15514900

O 1.85729500 -0.62264600 -1.21814400

H 1.22326100 -1.16899200 -0.40335400

H 1.18740500 -0.01374400 -1.59511500

O 1.13407700 1.04875400 1.07507100

H 0.59680600 1.83691100 0.80463700

H 1.24359100 1.12789200 2.02616700

S -1.57929500 -0.17884300 -0.51069700

O -0.53027400 0.46581900 -1.29375000

O -2.21604100 -1.32405600 -1.09755800

H -0.54992400 2.95621100 -0.77843800

H -1.43603300 2.73919700 0.56552000

N -0.54074500 3.08538300 0.22902300

H -0.48545900 4.07934200 0.41992200

SO_3_-2HONO-H_2_O-NH_3_-1:

N -0.69776700 -1.35908600 0.68936200

O -1.34044700 -1.83392100 1.53777400

O -2.18063200 0.90530500 0.44383500

O 0.46098800 -1.92455800 0.44571300

N 3.11065500 0.26876500 -0.53268900

O 3.77709200 -0.44396800 0.08801400

O 2.05667200 -0.44666000 -1.14754700

H 1.00059600 -1.30771700 -0.12052100

H 1.46002500 0.23227200 -1.50644000

O 0.93111100 0.64871300 1.30780400

H 0.62778400 1.56952100 1.12917400

H 1.22838600 0.64085300 2.22053300

S -1.61367700 0.09478900 -0.59350300

O -0.43125900 0.58611900 -1.26580200

O -2.42381100 -0.87050800 -1.27292000

H -0.11687000 3.11458700 -0.41226800

H -1.12266600 2.97436300 0.86442900

N -0.15276500 3.12532000 0.60242400

H 0.12299100 4.05032300 0.91250200

RC2-2W-A:

N -1.76491300 -0.28055100 0.71052600

O -2.61032000 -0.50973500 -0.12927200

O -0.95028000 -1.20555800 1.06609200

O -1.62284900 0.83866600 1.22811100

N 0.36747900 2.02041600 0.65592400

O 0.62809000 1.90086800 -0.36712300

O 1.26877900 0.36525700 1.89086400

H 1.65255600 -0.39070200 1.42160900

H 0.42324700 0.01363300 2.20729300

O -1.56250000 1.76664000 -1.65915300

H -2.35354800 1.56472000 -1.14639200

H -1.28868600 0.90447300 -1.99388300

S 0.30431900 -1.81746400 -0.69563100

O 1.53750300 -1.87363100 0.07951800

O 0.13545300 -0.55846800 -1.41327100

N 3.09543300 0.96535300 -0.44127800

H 3.66061200 1.01995400 0.40076700

H 3.68471400 1.25535800 -1.21459800

H 2.87499700 -0.01686200 -0.58702800

TS2-2W-A:

N -1.71527900 -1.18573600 -0.54581700

O -2.71774600 -1.38841600 0.07128900

O -1.40408300 0.76096600 -1.28897100

O -0.75415500 -1.94813400 -0.55858000

N 1.35116600 -1.64378800 0.53549800

O 1.41307600 -0.79381600 1.17010400

O 1.72773700 -0.97009500 -1.59532000

H 1.47306400 -0.03873100 -1.49067400

H 0.92454400 -1.40469800 -1.91272200

O -0.86271800 -0.36835900 2.12628600

H -1.69305800 -0.84790600 2.02824200

H -1.11249300 0.56591400 2.12406400

S -0.61195300 1.68243100 -0.43339300

O 0.81595100 1.29865100 -0.32610000

O -1.21779400 2.13987600 0.81339800

N 3.61883700 0.39937900 0.35384500

H 3.88366200 0.06224000 -0.56717400

H 4.46981500 0.67160700 0.83304200

H 3.06050400 1.23832500 0.21799100

IM2-2W-A:

N -1.35906400 0.51489100 0.85432100

O -2.45177100 0.99348800 0.89853300

O -1.84302700 -2.01751500 0.30327800

O -0.39559700 0.88455800 1.50881500

N 1.71846900 1.41639200 0.47829700

O 1.76740100 1.12515200 -0.54092000

O 2.17390900 -0.51099500 1.68958500

H 1.47816300 -0.97493000 1.18818100

H 1.79086300 -0.37039700 2.56131300

O -0.43625500 2.12968600 -1.36101200

H -1.08183500 2.83239100 -1.25313900

H -0.91989900 1.39688900 -1.77346900

S -1.09641200 -0.97703900 -0.34593300

O 0.36752400 -1.06708800 -0.26738500

O -1.60164200 -0.41467700 -1.58242600

N 3.28760900 -0.86846700 -0.96625200

H 3.66101500 -1.19633100 -0.08055800

H 3.97748100 -1.04852400 -1.68665600

H 2.46336600 -1.42705600 -1.17305800

IM2'-2W-A:

N 0.56014900 -0.33320800 1.27179500

O 1.48942800 0.34261900 1.63993100

O 2.07924300 -1.13338600 -0.72000300

O -0.48112100 -0.47056000 1.87539800

N -1.81082400 1.10004400 -0.15306800

O -2.35456800 1.46424900 -1.01680900

O 2.22211600 1.85870300 -0.78671000

H 2.09158400 1.26188700 -1.53179800

H 2.64083200 1.29274400 -0.12609200

O -2.73813200 -1.22222400 0.09608300

H -2.21717600 -1.32642700 0.90239100

H -2.30851200 -1.84224000 -0.50442300

S 0.65610300 -1.10422700 -0.49764800

O -0.06694000 -0.03758200 -1.20515100

O -0.07002400 -2.33743900 -0.31609000

N -0.33925500 2.46333100 0.44929700

H 0.52764300 2.25428000 -0.07367500

H -0.17800300 2.25114600 1.43193500

H -0.56453000 3.45005300 0.35468000

TS2'-2W-A:

N 1.07264900 0.52584400 1.01126400

O 2.01733400 1.17230100 1.26431700

O 2.53420600 -0.80373900 -0.75183000

O -0.02449500 0.72935300 1.62923900

N -2.86630200 -0.48246800 0.00224500

O -2.71183100 -1.02998300 -0.96172500

O 0.74684400 2.26063300 -1.28743600

H 0.69343500 1.46456300 -1.82913000

H 1.54803400 2.72099800 -1.54575600

O -1.72849900 -0.90176100 1.10056700

H -0.84492000 -0.00681400 1.33222700

H -1.23445500 -1.67314400 0.76098500

S 1.15693400 -0.94813000 -0.39564200

O 0.14094100 -0.44206400 -1.29659900

O 0.74512200 -2.04188200 0.45870400

N -2.00282600 1.96936900 -0.07656800

H -1.09101300 2.24213400 -0.44126700

H -2.05974200 2.32688500 0.87207800

H -2.70391300 2.46337700 -0.61933200

SO_3_-HONO-NH_4_NO_2_-H_2_O-2:

N 1.65663700 -0.75426100 1.02908000

O 2.48323600 -1.57476000 1.27036600

O 1.97235400 -1.24192700 -1.54593300

O 1.19324400 0.04792300 1.83019300

N -3.64877400 -0.25255000 0.16998200

O -3.84973800 -1.31935100 -0.26026400

O 0.82872200 3.39459500 -0.88426100

H 1.06554400 2.54440300 -1.28505500

H 0.89321100 4.07218900 -1.55921900

O -2.31253700 0.06978000 0.23100800

H 0.14432700 1.44677000 1.38627200

H -1.74247900 -0.67467400 -0.10969600

S 0.91743400 -0.70108300 -0.74469700

O 0.64351900 0.73395100 -0.83882200

O -0.28346300 -1.50587700 -0.53958900

N -0.58114400 2.14347500 1.12952300

H -0.17778000 2.77463900 0.40892900

H -0.89167700 2.66062700 1.94782200

H -1.35981200 1.60205600 0.72442500

t-ONONO_2_-H_2_O-(NH_3_)_2_:

N -0.04635400 1.31152400 -0.02336700

O 1.00121700 1.66564400 0.51342200

O -1.12253800 1.84064600 0.20044300

O -0.00414400 0.31439700 -0.83852200

N 0.80776400 -1.16834900 0.24834900

O 0.80070500 -2.05633000 -0.37791100

O 3.00867900 -0.14177900 -0.40186500

H 2.48566800 0.64982700 -0.19595100

H 3.89248600 0.03320100 -0.07399000

N -1.11588100 -1.07771000 1.49295500

H -1.85871400 -1.03611700 0.78130200

H -1.09091200 -0.17881200 1.96692300

H -1.33581500 -1.78997000 2.18110400

N -2.88503300 -0.47811800 -0.85710000

H -2.37231600 -0.73889000 -1.69307300

H -2.64106300 0.49522600 -0.68272900

H -3.87415700 -0.52651400 -1.07398700

c-ONONO_2_-H_2_O-(NH_3_)_2_:

N 0.68887300 -0.97928700 -0.61522800

O 1.74160100 -1.58488100 -0.52201400

O 0.28448900 -0.49980100 -1.68353100

O -0.01948600 -0.79365200 0.43467300

N -1.28735300 1.11334200 0.19855100

O -1.89212600 0.98032900 -0.69850000

H 0.45209300 3.00466900 -0.32161600

H 0.66308800 1.61134200 -1.23602200

O -2.67995400 -1.00520100 1.05689100

H -3.12500900 -1.68310900 1.56701300

H -1.77620500 -1.32133900 0.90772200

H 2.86944400 0.93441000 2.26249900

H 1.67028000 -0.08573500 1.77139400

N 2.35515500 0.59877100 1.45579700

N 0.47041900 1.99000100 -0.30912200

H 1.17721300 1.63166600 0.36427100

H 3.00324900 0.07394600 0.87459000

RC1-W-2A:

N 0.80490000 -1.26491300 -0.73468600

O 1.64067900 -1.96462800 -1.27099100

O 0.00126800 -1.74971000 0.11188100

O 0.73688700 -0.03392900 -0.97276600

N 0.23569700 1.70955800 0.62055400

O 1.01515500 2.43978200 0.34481100

O -0.80546800 2.17398900 -1.90318000

H -0.21959200 1.45069400 -2.15356100

H -1.59753200 1.70366500 -1.61969500

H 0.34128000 -0.36957400 1.67193900

H 1.91857300 0.23588800 1.47248200

S -2.27103700 -0.83004200 0.37367900

O -1.89075600 -0.14208100 1.59316800

O -2.41268100 0.00399100 -0.80021200

N 3.40455800 -0.21861300 0.40616200

H 3.47352100 -1.23156200 0.34182800

H 3.14586900 0.08559500 -0.53106900

H 4.33732100 0.13117800 0.59898900

N 0.94892700 0.44183300 1.80220500

H 0.89790700 0.78042700 2.75889200

TS1-W-2A:

N -1.18840300 0.16403000 1.26339400

O -1.93677100 -0.32540400 2.05231600

O -0.29265300 -1.31956700 0.17035800

O -1.03590800 1.35044700 1.06747000

N 0.50225100 1.46213800 -0.60796900

O 0.81901600 2.48934700 -0.79505200

O 2.08393900 1.26249100 1.43363700

H 1.64982000 1.32454600 2.28792500

H 2.40047200 0.34954000 1.37421900

H -0.43460000 0.23929000 -2.49049000

H -1.72691400 0.39247000 -1.43633800

S 1.11264900 -1.48191500 -0.37154000

O 1.38345800 -0.68091200 -1.57973600

O 2.16357000 -1.44864200 0.64946000

N -3.15964100 -0.76941700 -0.68484400

H -2.56870700 -1.51763900 -0.32938000

H -3.68631900 -0.42180100 0.11117800

H -3.82752400 -1.18293800 -1.32703800

N -0.97156900 0.89674000 -1.93061000

H -1.36427600 1.63067100 -2.51285700

IM1-W-2A:

N -0.72762700 -0.01937900 1.13859700

O -1.66400600 0.10783400 1.87421000

O -2.41596100 -0.85312700 -0.68219800

O 0.39822100 0.39045400 1.36396300

N 2.18190600 0.57380000 -0.22778700

O 2.92935900 0.88267000 0.50177500

O 2.42906800 -1.94514000 0.10574400

H 2.75126500 -2.64621900 0.67546600

H 1.50177700 -2.17136100 -0.07305700

H 0.96512400 1.80901000 -1.89717900

H 0.24114400 2.18969900 -0.47216400

S -0.98570400 -0.94922100 -0.54729700

O -0.17960000 -0.10904800 -1.41871400

O -0.40024300 -2.23480700 -0.20598500

N -1.72100400 2.40982900 -0.06533200

H -2.23968400 1.64100800 -0.48998100

H -1.98960700 2.41787800 0.91540700

H -2.08752600 3.26325700 -0.47565300

N 1.15734200 2.13983000 -0.95552300

H 1.61975900 3.04501100 -0.96612400

IM1'-W-2A:

N -0.72838500 -0.01922100 1.13803200

O -1.66498200 0.10837700 1.87330900

O -2.41637200 -0.85818000 -0.68077600

O 0.39728700 0.39113200 1.36339700

N 2.18095600 0.57403500 -0.22777400

O 2.92899900 0.88371900 0.50090700

O 2.43186400 -1.94422200 0.10768900

H 2.75156000 -2.64357200 0.68103200

H 1.50481900 -2.17000700 -0.07298300

H 0.96123900 1.80534500 -1.89652900

H 0.24207800 2.19215500 -0.47081000

S -0.98580700 -0.95033500 -0.54729400

O -0.18284900 -0.10819100 -1.41976500

O -0.39647700 -2.23420400 -0.20616100

N -1.71992600 2.41235600 -0.06427100

H -2.23725800 1.64423700 -0.49175300

H -1.98920800 2.41687400 0.91630700

H -2.08741400 3.26657600 -0.47207100

N 1.15682300 2.13899000 -0.95655300

H 1.62106300 3.04318500 -0.97132200

TS1'-W-2A:

N -0.76192500 0.08510300 1.19082900

O -1.48842500 0.66683100 1.91327000

O -2.62456300 0.11801600 -0.69714900

O 0.45700300 -0.06510600 1.50573000

N 3.24357800 -0.43497000 -0.29277300

O 3.66560900 -0.09664600 0.68785500

O 1.87594300 -1.26608600 -0.02087500

H 1.11773300 -0.66110400 0.72839100

H 1.40006000 -1.24944300 -0.87259200

H 1.38636400 1.10900400 -2.10590700

H 0.89897200 1.79877800 -0.72242600

S -1.51070700 -0.76860000 -0.51471300

O -0.35033500 -0.56991700 -1.36611200

O -1.72317400 -2.08958000 0.00536500

N -0.99626800 2.70514200 -0.14613000

H -1.82496700 2.19640900 -0.44706400

H -1.16733300 3.00059800 0.81046100

H -0.94695400 3.54784200 -0.70979900

N 1.72934200 1.45991300 -1.21626300

H 2.30790600 2.26911400 -1.41996700

SO_3_-HONO-NH_4_NO_2_-NH_3_-1:

N -1.86110800 -0.53050800 1.05920600

O -2.80116700 -1.23600800 1.24474600

O -1.82628900 1.00542900 -1.04543100

O -1.26236400 0.10308600 1.91710800

N 3.05470500 -0.63536100 0.14156300

O 4.20208400 -0.80531600 0.33107200

O 2.53169500 -1.49353900 -0.74272600

H 0.30686100 0.98932400 1.61726000

H 1.58551700 -1.22539600 -0.79755100

H 1.83064800 0.80131800 0.98789100

H 0.89943400 2.16673300 0.53534200

S -1.24945600 -0.29191200 -0.74376500

O 0.20218500 -0.24038700 -0.48834900

O -1.74774000 -1.45751300 -1.40934100

N 0.33939200 3.02122500 -0.79186500

H -0.55460500 2.56371400 -0.98482600

H 0.17138300 4.01998200 -0.72387700

H 0.92173900 2.87206800 -1.61097800

N 1.16492000 1.49858000 1.34570600

H 1.55771800 1.99928300 2.13807300

RC2-W-2A:

N 1.07111000 -1.08089500 0.65453100

O 2.00333800 -1.65957300 1.17388100

O 0.37287100 -1.65969800 -0.23666300

O 0.78387100 0.10007200 0.94492300

N 0.13587100 1.97603100 -0.48665600

O -0.95134400 2.05391300 -0.61624400

H 0.86501400 1.24811600 -2.72161800

H 0.33586400 -0.03312600 -1.77612700

O -0.89213900 1.93458500 2.23098300

H -0.07670200 1.44443000 2.37886600

H -1.50519700 1.23664400 1.97126100

S -1.92898600 -1.19992400 -0.33362700

O -1.76702000 -0.42879100 -1.55707300

O -2.11614200 -0.40563900 0.86467400

H 4.30410900 0.82338600 -0.70649900

H 3.17646000 0.59278100 0.46375600

N 3.44137400 0.33723700 -0.48579900

N 0.89108300 0.82568400 -1.79760800

H 1.86178500 0.65644800 -1.45564000

H 3.65889200 -0.65524600 -0.44309500

TS2-W-2A:

N 1.15249800 -0.45212700 1.23295700

O 2.03878800 -0.99461000 1.82077300

O 0.39700600 -1.52474100 -0.37813500

O 0.63828500 0.60485300 1.56504100

N -0.10692700 2.40980200 0.07637500

O -1.17045000 2.29543200 -0.15384500

H 1.26507600 2.39386500 -1.95815600

H 0.27590900 1.03219000 -1.84200700

O -2.27519400 0.73631300 1.59984800

H -1.50114500 0.47762700 2.10982000

H -2.60324500 -0.09302400 1.23039800

S -1.01368600 -1.35370200 -0.85557900

O -1.24328800 -0.09230200 -1.58984300

O -2.05635100 -1.72714000 0.09834900

H 3.90312800 -0.23379600 -1.54292000

H 3.64440500 -0.19628100 0.07162300

N 3.18031000 -0.18591300 -0.83223400

N 0.94577400 1.63811800 -1.35850400

H 1.75961800 1.07682100 -1.02859200

H 2.64327200 -1.04976800 -0.87855000

IM2-W-2A:

N 0.72481100 -0.26618700 1.10396900

O 1.70704300 -0.48652400 1.75701900

O 2.07431900 -0.82994400 -1.06621200

O -0.24389000 0.36136900 1.48583200

N -2.11541200 1.41698400 0.33238200

O -2.57888100 0.73121000 -0.37419700

H -0.95783800 3.48045400 -0.56655600

H -0.69029800 2.12672800 -1.53576400

O -2.48092500 -1.72398300 0.52306700

H -2.48270800 -2.12813200 1.39339400

H -1.71544300 -2.11442000 0.07651200

S 0.68585200 -0.96765100 -0.69840800

O -0.24832400 -0.03000400 -1.30908100

O 0.16446200 -2.29915900 -0.45313100

H 2.66204200 2.88097900 -0.15817300

H 2.39259800 1.87954500 1.10022700

N 2.12578300 2.07129200 0.13766200

N -0.73389800 2.48971800 -0.58534900

H 0.19492600 2.31206600 -0.15228600

H 2.46366500 1.27883100 -0.40984000

IM2'-W-2A:

N 0.44535600 -0.46948200 1.29293600

O 1.40512200 0.12985900 1.71320200

O 1.98281800 -1.38630800 -0.63304600

O -0.62652900 -0.54369300 1.85317100

N -1.62839100 1.31155800 -0.13383200

O -2.08431500 1.77773700 -1.00607800

H 0.01422500 2.11978000 1.54204300

H -0.30946200 3.43697100 0.58219600

O -2.86412800 -0.93283300 -0.08064100

H -2.39038200 -1.12535500 0.73824900

H -2.46198500 -1.55638700 -0.69584800

S 0.56391600 -1.18302200 -0.49877100

O 0.02353800 -0.00596700 -1.19268700

O -0.31956100 -2.32164500 -0.41892800

H 2.06504500 1.32412900 -1.65316700

H 3.11812000 2.39049900 -0.98106300

N 2.34222100 1.76801300 -0.78175400

N -0.11101600 2.43956500 0.58300100

H 0.77017900 2.24104300 0.04348300

H 2.69885100 1.01288300 -0.19796100

TS2'-W-2A:

N 0.94306100 0.11438500 1.20329600

O 1.95786600 0.57757400 1.57388400

O 2.27436200 -0.95063800 -0.82492500

O -0.10701900 0.25077400 1.91333800

N -2.89894300 0.19300600 -0.10363500

O -2.89101000 -0.19206500 -1.15152300

H -1.18407900 2.53917700 1.02661500

H -1.70845200 2.93756000 -0.46279600

O -2.02705600 -0.79507800 0.89418500

H -1.02379600 -0.22026800 1.41209100

H -1.68284300 -1.54782900 0.37783000

S 0.87471100 -1.02256700 -0.52605900

O -0.05266000 -0.24139800 -1.30779000

O 0.33408600 -2.20958200 0.10081800

H 1.50969600 1.80954800 -1.99827700

H 2.12568400 3.08268700 -1.17215100

N 1.75784900 2.14243900 -1.07117100

N -1.25429800 2.20273500 0.07089600

H -0.29982800 2.11992200 -0.29003100

H 2.52602400 1.55563300 -0.75593600

SO_3_-HONO-NH_4_NO_2_-NH_3_-2:

N -2.19303500 0.64832800 -0.24843600

O -3.36333200 0.43106700 -0.23475500

O -1.81540600 -1.43590200 1.32207200

O -1.66239900 1.66688700 -0.67189100

N 2.69634300 -0.85512400 -0.62320400

O 2.37329000 -1.72579800 0.09125500

H 1.34298500 3.02367900 -1.10066900

H 1.41936900 1.35962900 -1.32250500

O 1.78187000 -0.56166600 -1.59487500

H 0.13837600 2.00194800 -0.50007600

H 0.94165500 -1.07525100 -1.42886800

S -1.00213200 -0.72162000 0.38596500

O 0.07334600 0.12672900 0.89726000

O -0.67433200 -1.37116600 -0.88353200

H 1.78289800 0.76973100 1.95644300

H 3.34692200 0.92183400 1.48952300

N 2.48829000 1.44016200 1.65139300

N 1.15092100 2.11731200 -0.68307900

H 1.69747800 1.96644500 0.23169300

H 2.66249600 2.08194000 2.41802100
